# Selective TRPV2 Antagonists Derived from the Natural Product Piperlongumine Inhibit Cancer Cell Migration and Metastasis

**DOI:** 10.1021/acschembio.5c00935

**Published:** 2026-03-25

**Authors:** Hannah Kiely-Collins, Cong Tang, Marta C. Marques, Laura Butron, Angela Lamberti, Nicholas Bossons, Fabian Offensperger, Vincenth Brennsteiner, Bárbara Sousa, Luís Carvalho, Antonio Ferrer-Montiel, Francisco Corzana, Georg E. Winter, Asia Fernandez-Carvajal, Gonçalo J. L. Bernardes

**Affiliations:** † Yusuf Hamied Department of Chemistry, 2152 University of Cambridge, Lensfield Road, Cambridge CB2 1EW, U.K.; ‡ GIMM - Gulbenkian Institute for Molecular Medicine, Avenida Prof. Egas Moniz, Lisboa 1649-028, Portugal; § Instituto de Investigación, Desarrollo e Innovación en Biotecnología Sanitaria de Elche (IDiBE), Universidad Miguel Hernandez, Avda. Universidad s/n, Elche 03202, Spain; ∥ Chemprecise Lda, Torres Vedras 2560-247, Portugal; ⊥ Research Institute for Medicines (iMed.ULisboa), Faculty of Pharmacy, Universidade de Lisboa, Lisbon 1649-003, Portugal; # CeMM Research Center for Molecular Medicine of the Austrian Academy of Sciences, Vienna 1090, Austria; ∇ Departamento de Química, Universidad de La Rioja, Instituto de Investigación en Química (IQUR), Logroño 26006, Spain; ○ Translational Chemical Biology Group, Spanish National Cancer Research Centre 26 (CNIO), Madrid 28029, Spain

## Abstract

TRPV2 is the least studied member of the vanilloid TRP subfamily despite its emerging relevance in cancer metastasis, pain, and inflammation. Although several small-molecule TRPV2 modulators have been reported, including the natural products piperlongumine (PL) and cannabidiol, all lack selectivity, complicating the interpretation of phenotypic readouts and functional insights into the role of the channel in health and disease. Here, we report a series of PL-based derivatives rationally designed to maintain TRPV2 antagonism while eliminating covalent off-target activity associated with the electrophilic groups present in PL. Using electrophysiological and calcium fluorescence imaging assays in HEK293T cells and DRG nociceptors, we identified HKC54 as the most potent TRPV2 antagonist to date (IC_50_ = 0.4 μM), displaying ∼50-fold selectivity over TRPV1 and ∼70-fold selectivity over TRPA1. Cellular thermal shift assays demonstrated direct TRPV2 engagement, and molecular dynamics and docking studies suggest a near-identical binding mode of the derivatives to PL. To assess proteome-wide selectivity, we pursued an unbiased chemoproteomic strategy and developed photoaffinity probes derived from PL and noncovalent derivative HKC22. Whereas the PL-based probe labeled many established covalent and noncovalent PL targets (e.g., GSTP1, GSTO1, STAT3, and KEAP1), no off-targets were detected for HKC22, suggesting high selectivity for TRPV2. Finally, PL derivatives inhibited cancer cell migration in vitro and suppressed metastasis in vivo, underscoring the therapeutic potential of selective TRPV2 antagonists.

## Graphical abstract

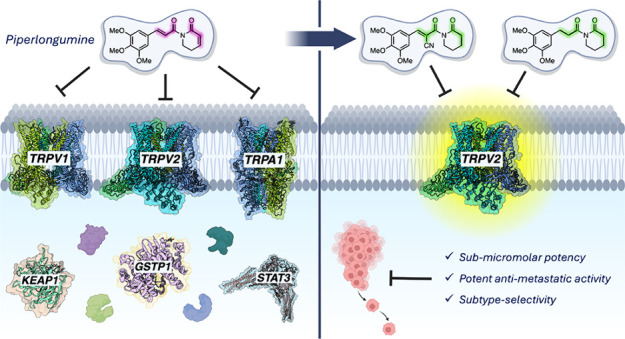

## Introduction

Transient receptor potential (TRP) channels are a superfamily of membrane proteins that contribute to calcium homeostasis. With over 50 characterized members, they constitute the second largest family of voltage-gated-like ion channels after the potassium superfamily.^1^ Members share a conserved membrane topology of six transmembrane segments (S1–S6), with a pore-forming loop between S5 and S6. The N- and C-terminal tails are cytoplasmic.^2^ The mammalian TRPV (vanilloid) subfamily comprises six members (TRPV1–6) grouped into thermosensitive, calcium-nonselective TRPV1–4, and calcium-selective TRPV5–6. TRPV1 is the best studied owing to its critical role in thermosensation and nociception and the discovery that capsaicin directly activates the channel. In contrast, TRPV2 is not activated by capsaicin, despite sharing ∼50% sequence identity with TRPV1, and remains the least characterized TRPV member at the molecular level.^2^ TRPV2 is activated by physical stimuli including heat (> 52 °C), mechanical stretch, and osmotic swelling.^2^

TRPV2 is broadly expressed in the brain, vascular smooth muscle cells, gastrointestinal tract, macrophages, and urothelial tract. Correspondingly, it is known to play vital roles in neuronal development, cardiac function, and immunity.^3^ Aberrant expression of TRPV2 has been implicated in muscular dystrophy,^4^ cardiomyopathy,^5^ diabetes,^6^ and multiple cancers.^7,8^ Notably, TRPV2 appears to drive cancer cell invasion and metastasis rather than proliferation. Despite its important roles in physiology and disease, few endogenous or synthetic chemical modulators of TRPV2 are known, and most lack selectivity across the TRP superfamily, complicating interpretation of phenotypic readouts in systems that coexpress several TRP channels.^2^

Cannabinoids, including (−)-*trans-*Δ^9^-tetrahydrocannabinol, cannabidiol (CBD), and δ^9^-tetrahydrocannabivarin, are among the most potent TRPV2 agonists but activate several other TRPV2 channels; CBD is also a potent activator of TRPV1 and TRPA1.^2^ Additional nonselective agonists include probenecid,^9–11^ 2-aminoethoxydiphenyl borate,^11–13^ and nonpsychotropic cannabinoid Δ^9^-tetra-hydrocannabiorcol (C16).^11^ Among antagonists, ruthenium red is potent but broadly nonselective.^11,13,14^ Tranilast and its derivatives^5^ have been used as TRPV2-specific antagonists, but direct, selective antagonism remains insufficiently validated.^2^ Valdecoxib^15^ and SET2 ^16^ were purported to be subtype-selective TRPV2 antagonists, yet their proteome-wide selectivity has not been profiled. These limitations underscore the need for selective TRPV2 modulators and the systematic characterization of their proteome-wide targets.

Photoaffinity labeling (PAL) is a valuable approach for profiling ligand-protein interactions in the native cellular environment. Uniquely, by conjugating a photoreactive group and click handle to a ligand, PAL can capture both covalent and reversible transient interactions. When coupled with MS-based proteomics, PAL enables unbiased assessment of proteome-wide selectivity and direct target engagement. PAL has previously been used to profile TRPC3 and TRPC5 ligands,^17,18^ providing precedent for TRP-channel profiling, but no PAL probes targeting any TRPV member have been reported. To complement chemoproteomic approaches, orthogonal strategies, such as the cellular thermal shift assay (CETSA), are often employed to confirm target engagement. CETSA measures changes in protein thermal stability upon ligand binding, and although initially optimized for soluble cytosolic proteins, recent advances incorporating mild detergents now permit its application to membrane proteins, including multipass transmembrane proteins like TRPV2.^41,42^ Together, PAL and CETSA provide complementary approaches to validate both binding and selectivity of candidate TRPV2 antagonists in a physiologically relevant context.

We previously identified the alkaloid natural product piperlongumine (PL; piplartine) as an allosteric antagonist of TRPV2 using the machine learning platform SPiDER.^19^ A cryo-EM structure of PL bound to full-length rat TRPV2 (3.4 Å; PDB: 6WKN) revealed a noncovalent binding mode: the acyclic imide carbonyl forms key hydrogen bonds with T522 and R539, a methoxy substituent on the phenyl ring engages G530, and the Michael acceptors contribute hydrophobic contacts with Y525, V543, and L513.^19^ Although PL contains two electrophilic Michael acceptors and covalently modifies numerous cellular proteins including KEAP1,^20^ GSTO1,^21^ and STAT3,^22^ washout experiments demonstrated reversible TRPV2 inhibition, and no covalent adducts were evident in the cryo-EM structure. PL has potent anticancer and antiviral properties,^23–29^ but its electrophilicity drives extensive off-target covalent reactivity. Therefore, given that PL engages TRPV2 noncovalently, we hypothesized that PL derivatives lacking the electrophilic groups could retain TRPV2 antagonism while minimizing off-target interactions.

In this study, we report the design, synthesis, and characterization of PL derivatives that lack the key electrophilic groups that cause covalent off-targets yet maintain and improve TRPV2 antagonism. Using electrophysiological and calcium fluorescence imaging assays in both HEK293T cells and rat dorsal root ganglion (DRG) neurons, we identify HKC54 as the most potent TRPV2 antagonist described to date (IC_50_ = 0.4 μM), displaying ∼50-fold selectivity over TRPV1 and ∼70-fold selectivity over TRPA1. To characterize target engagement and proteome-wide selectivity, we developed PAL probes derived from PL and noncovalent derivative HKC22, the first PAL probe for any TRPV channel. Proteomic analysis revealed no detectable off-targets for HKC22, in stark contrast to the PL-based probe, which labeled numerous known covalent and noncovalent PL targets. CETSA confirmed direct target engagement of TRPV2 by HKC22 and HKC54, and molecular docking and dynamics provided insight into the near-identical binding mode of the analogues to PL. Finally, TRPV2 is emerging as an important antimetastasis target, and HKC22 and HKC54 exhibited potent inhibition of cellular migration in vitro and in vivo. Therefore, the PL derivatives presented herein represent a promising starting point for the development of selective TRPV2 antagonists that possess potent antimetastatic activity.

## Results

### Design of PL Derivatives to Remove Covalent Off-Targets

Since PL forms noncovalent interactions with TRPV2, we reasoned that removing or modifying the electrophilic groups of PL could both mitigate irreversible covalent off-targets and increase selectivity for TRPV2. The structure–activity relationship (SAR) of PL for its covalent targets is well-documented.^30^ However, the SAR for reversibly binding TRPV2 remains unexplored. To that end, we designed and synthesized a range of PL analogues with different degrees of unsaturation and electrophilicity (Figure 1A); HKC54 contains a cyanoacrylamide that can form reversible covalent protein interactions.^31^ Previous SAR studies have demonstrated that 2,3-olefin is particularly important for glutathione (GSH) depletion, reactive oxygen species (ROS) elevation, and cytotoxicity of PL.^21,28^ Moreover, small-molecule thiols undergo preferential heteroconjugation to C3 over C8 in vitro.^32^ Therefore, we prioritized analogues lacking the more reactive C2–C3 olefin. The C7–C8 olefin, though not as reactive as C2–C3, may be required for binding TRPV2 since it imposes some conformational rigidity, and the π bond itself may be important for the hydrophobic interactions formed with Y525, V543, and L513.^33^ To evaluate whether the derivatives had the same binding mode as PL, we performed molecular docking of HKC22 and HKC54 in the PL binding site of the PL-bound TRPV2 cryo-EM structure (Figure 1F). We observed a near-identical binding mode of the derivatives to PL, except that HKC22 and HKC54 appear to engage both of their carbonyl groups in hydrogen bonding interactions to R539, whereas only the acyclic carbonyl of PL engages in hydrogen bonds to R539 (and T522) (Figure S1A–C). To further model the binding mode and assess its stability, we performed a 300 ns molecular dynamics (MD) simulation of HKC22 bound to membrane-embedded TRPV2. The binding mode of the ligand remains stable, with key interactions mirroring those formed by PL (Figure S1D–F). These include a CH/π interaction between the aromatic ring of HKC22 and L513, along with hydrogen bonds between the exocyclic carbonyl group of the ligand and the hydroxyl side chain of T522, and between one of the aromatic methoxy groups and the amide −NH_2_ side chain of Q530. Taken together, the molecular docking and MD results suggest that the designed PL derivatives retain the same key interactions with TRPV2 even in the absence of one or both electrophilic groups. We next sought to evaluate whether the phenotypic effects of covalent off-targets, namely GSH depletion, ROS induction, and cytotoxicity,^28^ had been removed as a surrogate measure of enhanced selectivity for TRPV2.

**Figure 1:**
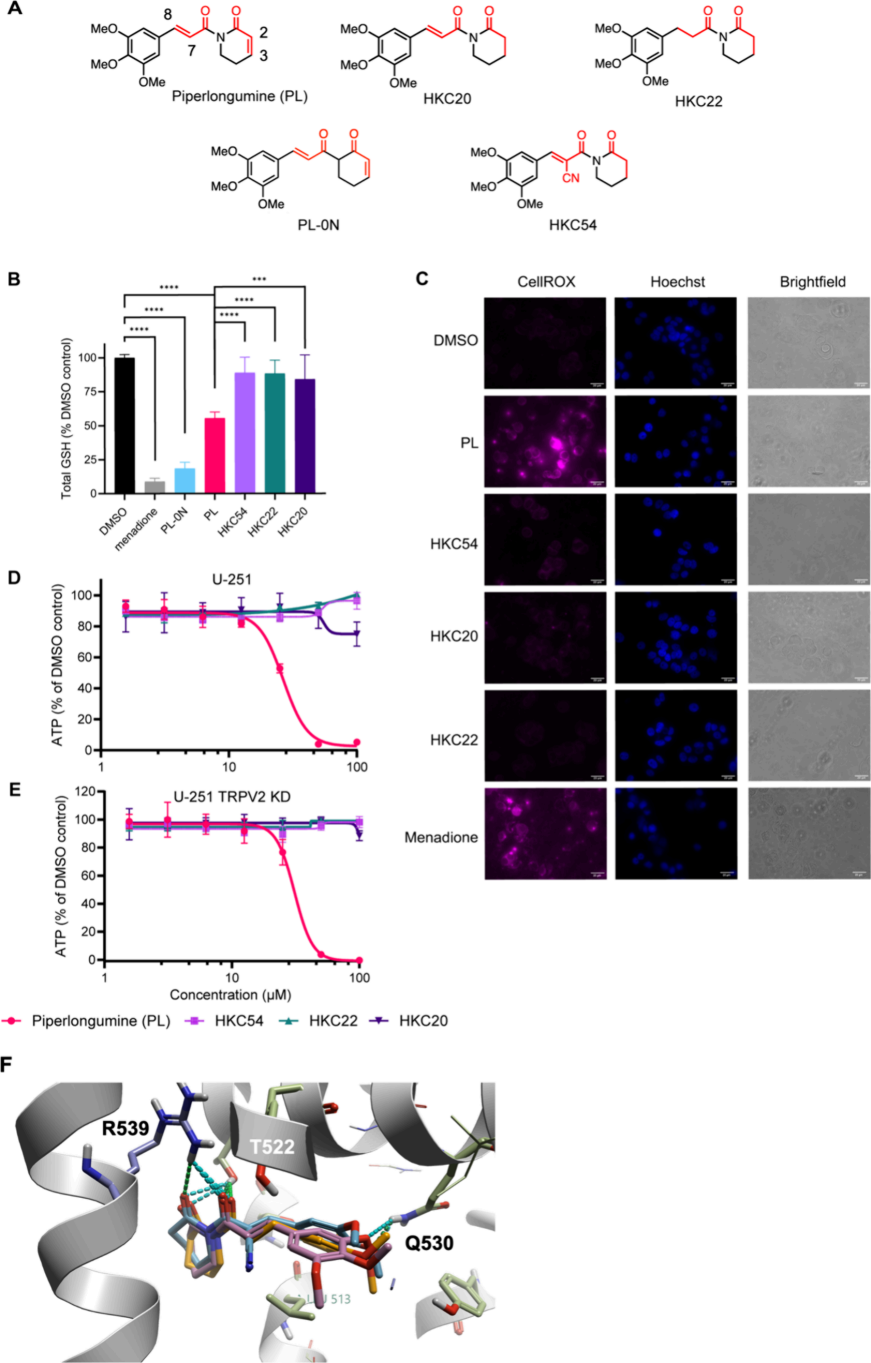
Evaluating the ability of PL and derivatives to deplete GSH and induce ROS. (A) Chemical structures of PL and derivatives HKC20, HKC22, HKC54, and PL-0N. Electrophilic Michael acceptors (or lack thereof) are highlighted in red. (B) Effect of PL and derivatives on GSH levels using the GSH/GSSG-Glo assay. MCF-7 cells were treated for 6 h with 20 μM of each compound (PL, HKC20, HKC22, HKC54, PL-0N, and menadione) or DMSO vehicle control. Data are presented as mean ± SD (*n* = 6). ****p < 0.0001, ***p < 0.001; one-way ANOVA, Tukey’s test. (C) Fluorescence imaging of ROS using CellROX Deep Red (5 μM) with Hoechst 33342 staining of nuclei. MCF-7 cells were treated for 4 h with 20 μM of each compound (PL, HKC20, HKC22, HKC54, and menadione) or the DMSO vehicle control. Representative images for each compound are shown. (D,E) Cell viability results of PL, HKC54, HKC22, and HKC20 treatment after 48 h, measured using CellTiter-Glo. GBM cell lines U-251 and U-251 TRPV2 KD, respectively. Data are shown as the mean ± SD (*n* = 3). (F) Molecular docking of PL analogues HKC22 and HKC54 in the PL-bound TRPV2 cryo-EM structure (PDB: 6WKN). PL (pink), HKC54 (blue), and HKC22 (orange). Key hydrogen bonding interactions displayed as green and blue dotted lines to key residues GLN 530, THR 522, and ARG 539.

### Confirming the Phenotypic Effects of Covalent Off-Target Removal

To compare the extent of GSH depletion caused by PL and derivatives, we measured total GSH levels using the GSH/GSSG-Glo assay following treatment of MCF-7 breast cancer cells for 6 h with 20 μM of each compound (menadione, PL-0N, PL, HKC54, HKC22, and HKC20) or vehicle control. We used the naphthoquinone menadione as a positive control since it is known to dramatically deplete GSH and induce the formation of ROS such as superoxide and hydrogen peroxide.^34,35^ None of the PL derivatives which lack the C2–C3 olefin (Figure 1A) significantly depleted the levels of total GSH (Figure 1B). PL depleted total GSH levels by ∼45% (*p* < 0.0001) compared to vehicle control and derivatives HKC54, HKC22, and HKC20. Treatment with PL-0N,^36^ which lacks the imide in PL and consequently has a more electrophilic C2–C3 olefin, led to a ∼80% reduction in total GSH, similar to positive control menadione which reduced levels by ∼90%. To evaluate the ability of PL and derivatives to induce ROS, we performed fluorescence imaging using a redox-sensitive dye, CellROX Deep Red, following treatment of MCF-7 cells for 4 h with 20 μM of each compound (menadione, PL, HKC54, HKC22, and HKC20) or vehicle control. Again, menadione was used as a positive control. Only PL and menadione induced visible ROS (Figure 1C), highlighting the importance of C2–C3 olefin for the induction of oxidative stress observed upon PL treatment. Collectively, the lack of GSH depletion and ROS induction suggest that removing the C2–C3 olefin abolishes the phenotypic effects of irreversible covalent off-target engagement even when the C7–C8 olefin is still present (HKC20) or modified (HKC54).

We next measured the effect of PL and derivatives on the cell viability of multiple cancer cell lines (Figures 1D,E and S2A–D). The removal of the electrophilic groups of PL has been shown to significantly diminish its cytotoxicity in multiple studies.^21,30^ We chose a range of GBM cell lines (U-251, U-251 TRPV2 KD, and U-87 MG) due to our prior study that investigated PL-mediated TRPV2 inhibition in the context of GBM.^19^ In addition, we selected two acute myeloid leukemia (AML) cell lines (MOLM-13 and HEL) since PL has demonstrated high potency in AML cells and selectivity over nontransformed cells.^37,38^ It is evident that only PL significantly reduces cell viability in all cell lines tested (Figures 1D,E and S2A–D). Having demonstrated that the characteristic phenotypic effects that rely on the electrophilic groups of PL were abolished upon Michael acceptor removal, we next sought to investigate whether the TRPV2 antagonism was crucially maintained by the PL derivatives.

### Noncovalent Piperlongumine Derivatives Maintain TRPV2 Antagonism

To evaluate the ability of PL derivatives to inhibit TRPV2-mediated Ca^2+^ influx, HEK293T cells were transfected with hTRPV2-RFP and loaded with the Ca^2+^-sensitive dye Fura-2 AM (5 μM, 1 h). As a negative control, cells were transfected with the RFP pcDNA3.1­(+) empty vector. Ratiometric imaging (*F*_340_/*F*_380_) revealed robust Ca^2+^ influx upon stimulation with agonist CBD (4 μM), which was markedly reduced following pretreatment with PL and derivatives (5 μM). All tested PL derivatives significantly inhibited CBD-evoked Ca^2+^ influx compared with PL itself, with HKC22 displaying the most significant inhibition (*p* < 0.0001; one-way ANOVA, Tukey’s test; Figure 2A,B). These results provide insight into the SAR of reversible TRPV2 inhibition and demonstrate that TRPV2 inhibition is crucially maintained and even significantly improved after removal of one (HKC20) or both (HKC22) of PL’s Michael acceptors. Moreover, an α-cyano group (HKC54) is tolerated and results in improved calcium influx inhibition relative to PL.

**Figure 2:**
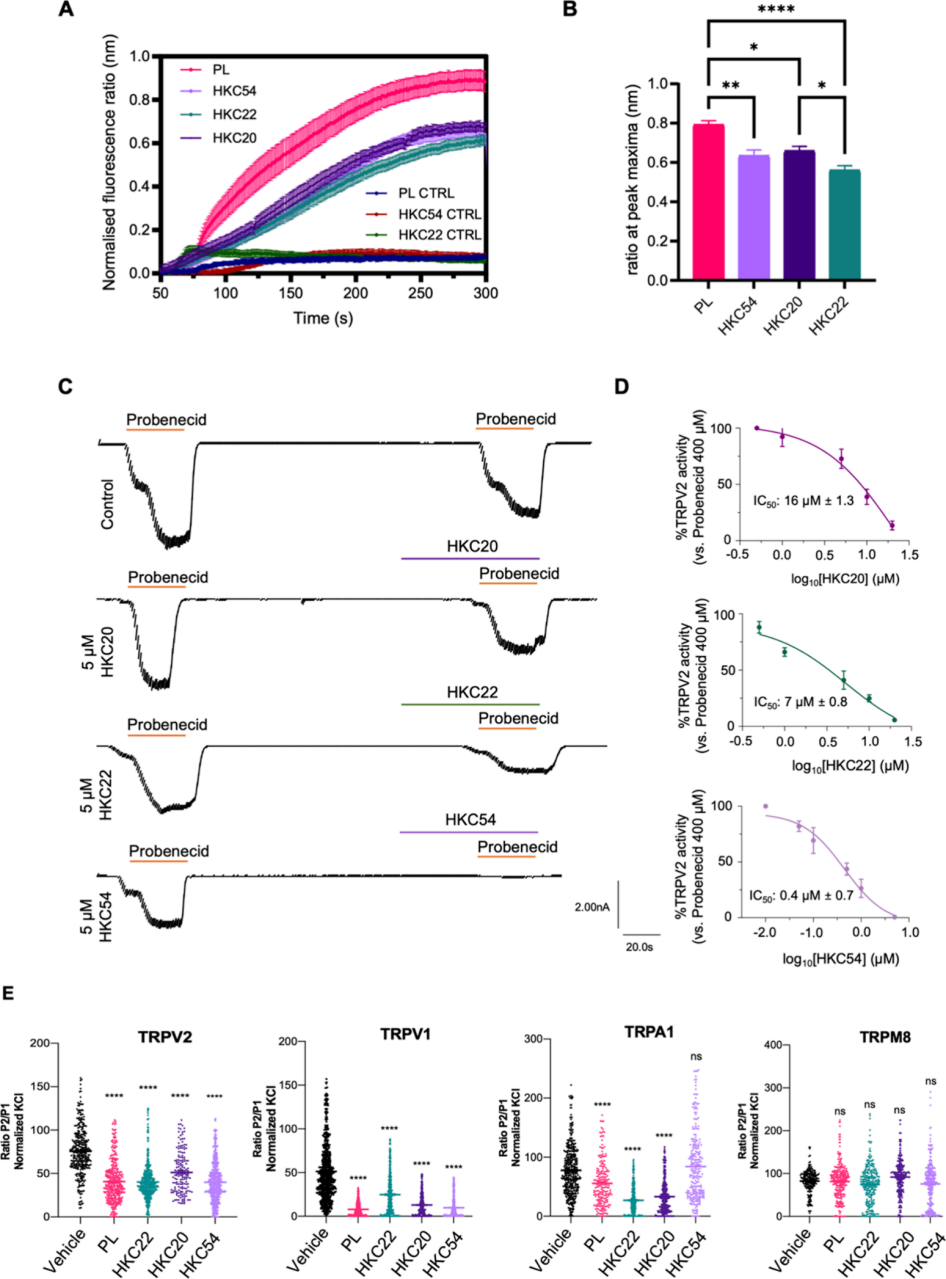
(A) Fura-2 AM calcium imaging in hTRPV2-RFP-transfected HEK293T cells. Control cells were transfected with the RFP pcDNA3.1­(+) empty vector. Representative ratiometric fluorescence traces (*F*_340_/*F*_380_) showing changes in intracellular Ca^2+^ concentration ([Ca^2+^]_i_) following loading with Ca^2+^-sensitive dye Fura-2 AM (5 μM, 1 h), treatment with PL and derivatives (5 μM), and stimulation with CBD (4 μM). For quantification, 40–60 responding cells were analyzed per well (*n* = 3), and fluorescence values were normalized to baseline. (B) Bar graph of peak maxima at 260s following treatment with PL and derivatives (5 μM) and poststimulation with CBD (4 μM). Data are presented as mean ± SEM ****p < 0.0001, **p < 0.01, *p < 0.05; one-way ANOVA, Tukey’s test. (C) Electrophysiological evaluation of PL derivatives on TRPV2 from rat DRG nociceptors. (D) TRPV2 patch clamp evaluation of PL derivatives quantified. (E) TRPV1: P2/P1 ratio denoting TRPV1 current evoked by each pulse of agonist capsaicin (0.1 μM), normalized to first vanilloid pulse, in the absence (control, *n* = 4) and presence of 10 μM PL, HKC22, HKC20, and HKC54 (*n* = 5) before the second pulse of capsaicin. Two consecutive agonist pulses (P1 and P2) are applied to account for channel desensitization. TRPA1: P2/P1 ratio denoting TRPA1 current evoked by each pulse of agonist AITC (100 μM), normalized to first vanilloid pulse, in the absence (control, *n* = 4) and presence of 10 μM PL, HKC22, HKC20, and HKC54 (*n* = 5) before the second pulse of AITC. Two consecutive agonist pulses (P1 and P2) are applied to account for channel desensitization. TRPM8: P2/P1 ratio denoting TRPM8 current evoked by each pulse of agonist menthol (100 μM), normalized to first vanilloid pulse, in the absence (control, *n* = 4) and presence of 10 μM PL, HKC22, HKC20, and HKC54 (*n* = 5) before the second pulse of menthol. Two consecutive agonist pulses (P1 and P2) are applied to account for channel desensitization. All data are expressed as mean ± SEM. Data were analyzed using an unpaired, two-tail Student’s *t* test. ****p < 0.0001.

To assess the activity of the PL derivatives under more physiologically relevant conditions, we next measured their effects on TRPV2 in primary cultures of rat neonatal DRG neurons in both electrophysiological and calcium fluorescence imaging experiments (Figure 2C,D). DRG neurons provide a native-like environment for the study of thermoTRP channels.^39^ In the electrophysiological recordings, all tested PL derivatives significantly inhibited the probenecid-evoked TRPV2 current at a fixed concentration (5 μM), with HKC54 exhibiting the greatest potency and effectively reducing the current to near baseline levels (Figure 2C). In the calcium fluorescence imaging assays, HKC20 and HKC22 produced clear concentration-dependent inhibition of probenecid-evoked Ca^2+^ influx, measured using Ca^2+^-sensitive dye Fluo-4 AM, with IC_50_ values of 16 and 7 μM, respectively (Figure 2D). Notably, HKC54 exhibited markedly higher potency with a submicromolar IC_50_ of 0.4 μM, representing the most potent TRPV2 antagonist reported to date (Figure 2D).

We next investigated the selectivity of the PL derivatives for TRPV2 over structurally homologous thermoTRP channels TRPV1 (the ‘capsaicin receptor’), TRPA1 (the ‘chemical nociceptor’ or 'wasabi receptor'), and TRPM8 (the ‘cold and menthol receptor’) by performing analogous calcium fluorescence imaging assays in DRG neurons (Figure 2E). The pore domain is highly conserved across all TRP-channel subfamilies, but the cytoplasmic N- and C-termini differ substantially.^40^ TRPV2 shares 50% sequence homology with TRPV1 but, unlike TRPV1, is not activated by capsaicin.^41^ The PL derivatives were initially tested at a fixed concentration (10 μM), at which all derivatives significantly inhibited calcium influx evoked by the TRPV1 agonist capsaicin, thus acting as TRPV1 antagonists (Figure 2E), with PL being the most potent. Similarly, PL and all derivatives except HKC54 significantly inhibited calcium influx induced by the TRPA1 agonist allyl isothiocyanate (AITC), with HKC22 being the most potent and PL the weakest antagonist (Figure 2E). No significant agonism or antagonism of TRPM8 was measured for any compound (Figure 2E). To further characterize these effects, we performed TRPV1 and TRPA1 dose–response calcium fluorescence imaging studies (Figures S3 and S4). HKC54 demonstrated approximately 50-fold and 70-fold selectivity for TRPV2 over TRPV1 and TRPA1, respectively, with IC_50_ values of 20.1 and 29.3 μM (Figures S3D and S4D). In contrast, HKC20 appears to be most potent against TRPV1 (IC_50_ = 1.7 μM; Figure S3C), and HKC22 is most potent against TRPA1 (IC_50_ = 1.1 μM; Figure S4B). Collectively, these results demonstrate that while PL and its derivatives retain some activity toward other thermoTRP channels, HKC54 exhibits markedly improved selectivity for TRPV2 over TRPV1 and TRPA1, establishing it as the most potent and selective TRPV2 antagonist in this series.

### Evidence of Direct TRPV2 Target Engagement by CETSA

To confirm the direct target engagement of TRPV2 by the PL derivatives, we performed CETSA (Figure 3A). Performing CETSA for membrane proteins had been challenging in the past because early protocols, optimized for soluble cytosolic targets, used detergent-free extraction buffers that precluded membrane protein recovery. The introduction of mild detergents in updated workflows now enables reliable detection of compound-induced changes in membrane protein thermal stability, including multipass transmembrane proteins such as TRPV2.^42,43^ To this end, we incubated MDA-MB-231 breast cancer cell lysates with 10 μM HKC22, HKC54, or vehicle control and subjected them to a temperature gradient between 45 and 85 °C in 5 °C increments. Immunoblot analysis revealed significant and reproducible thermal stabilization of TRPV2 upon treatment with HKC22 and HKC54 compared to vehicle control (Figures 3B and S5A).

**Figure 3:**
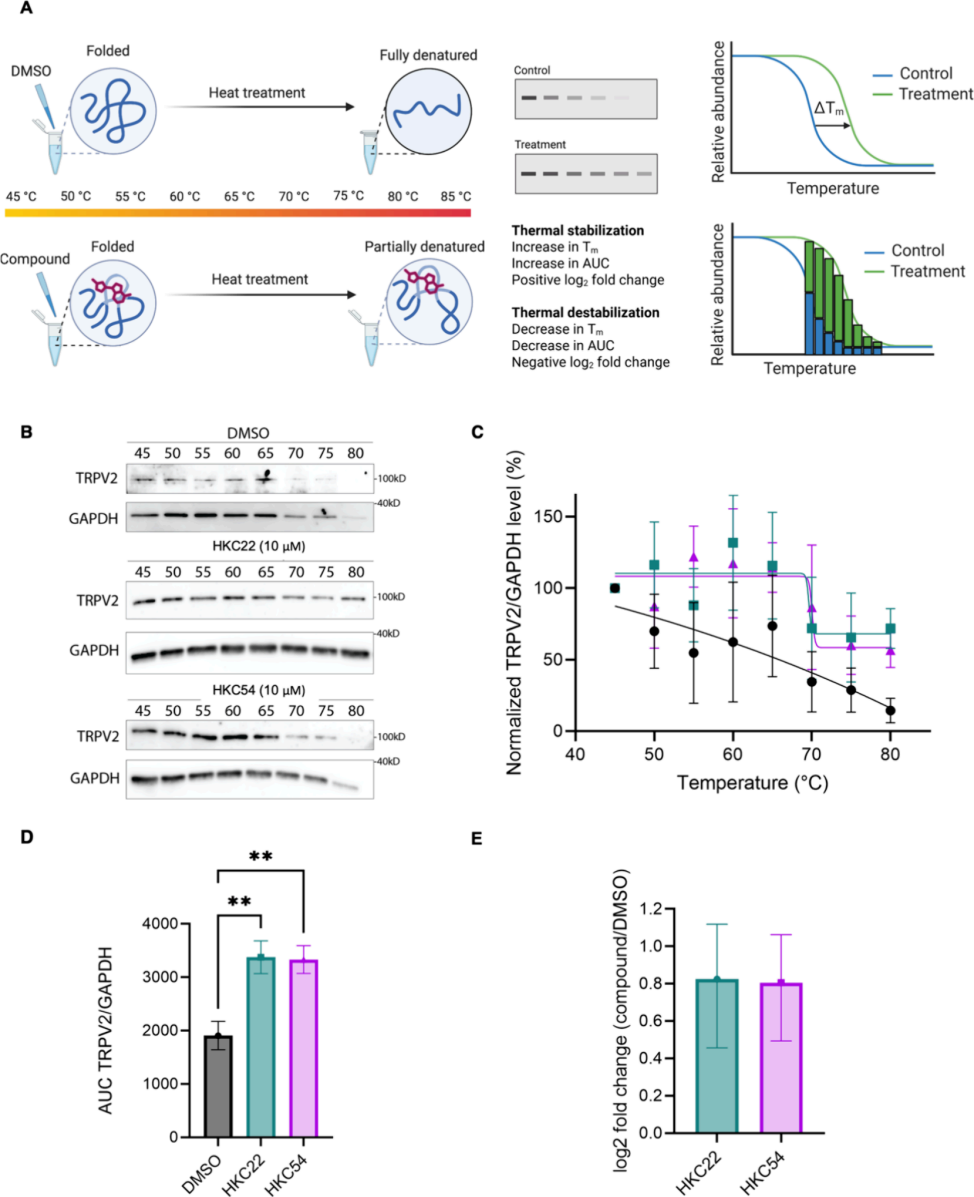
(A) Schematic of the CETSA workflow with compound treatment in lysate and analysis via immunoblotting. Protein stabilization can be quantified by the change in melting temperature (*T*_m_) between control and compound treatment for sigmoidal melt curves or by comparison of the relative AUC for nonsigmoidal melt curves. (B) Immunoblot CETSA results from treating MDA-MB-231 cell lysate with 10 μM HKC22, HKC54, and vehicle control. Subsequently, lysates were subjected to a temperature gradient between 45 and 80 °C, with steps every 5 °C. (C) CETSA TRPV2 protein level, normalized to GAPDH level, quantified (*n* = 3 of independent experiments, data presented as mean ± SEM). (D) Absolute AUC values of the TRPV2 level, normalized to the GAPDH level, following either vehicle (DMSO) or compound treatment with HKC22 and HKC54. Statistical significance was calculated with one-way ANOVA and Tukey’s multiple comparisons test. **p < 0.01. (E) Relative increase in AUCs of TRPV2 level following compound treatment with HKC22 and HKC54, denoting protein stabilization, presented as a log2 FC increase relative to DMSO.

To quantify this stabilization, we first normalized the remaining soluble TRPV2 fraction to GAPDH across the full temperature range (Figure 3C). Because membrane and phase-separating proteins often exhibit nonsigmoidal melting profiles, we quantified stabilization using an area under the curve (AUC)-based approach, which provides a more reliable measure of compound-induced thermal stabilization than relying solely on a *T*_m_-centric approach.^44,45^ HKC22 and HKC54 treatment significantly stabilized TRPV2 (*p* < 0.01; one-way ANOVA, Tukey’s test; Figure 3D), reflected by a log2 fold-change (FC) of 0.8 (approximately 1.7-fold increase) in AUC relative to vehicle control (Figure 3E). These results, together with the electrophysiological measurements and calcium fluorescence imaging assays (Figure 2), provide strong evidence of direct TRPV2 engagement by the PL derivatives. We next sought to profile the proteome-wide selectivity of the PL derivatives relative to PL by developing photoaffinity probes.

### Photoaffinity Labeling Demonstrates Proteome-Wide Selectivity of Noncovalent PL Derivative

To profile the proteome-wide targets of the noncovalent PL derivatives and assess their selectivity relative to PL, we developed photoaffinity probes derived from PL and derivative HKC22, named Photo-PL and Photo-HKC22, respectively (Figure 4B). To identify a suitable site for PAL linker conjugation that would preserve both binding interactions and bioactivity, we considered the cryo-EM structure of PL-bound TRPV2 (PDB: 6WKN), previous SAR studies of PL that focused on its covalent targets, and earlier reports describing PL-based activity-based probes (ABPs).^19^ Although ABPs can effectively capture direct covalent targets, they fail to identify transient and reversible interactions that can be captured by PAL probes.^46^ Consistent with this, previously reported PL-based ABPs did not label known noncovalent PL-binding proteins, such as TRPV2 ^19^ and GSTP1.^32^ All reported PL-based ABPs were functionalized at the *para*-position of the phenyl ring, which appears solvent-exposed in the PL-bound TRPV2 structure (Figure 4A). We therefore conjugated a “minimalist” PAL linker to this position, comprising an alkyl diazirine photoreactive group and a bioorthogonal alkyne handle for downstream click chemistry, to form Photo-PL and Photo-HKC22 (Figure 4B).^47,48^ Importantly, we retained the trimethoxyphenyl substituents absent in previously reported ABP **PL-5**, as it is a key pharmacophore for both the microtubule-destabilizing activity of PL^49^ and the noncovalent TRPV2 inhibition mediated by PL (Figure S1A).

**Figure 4:**
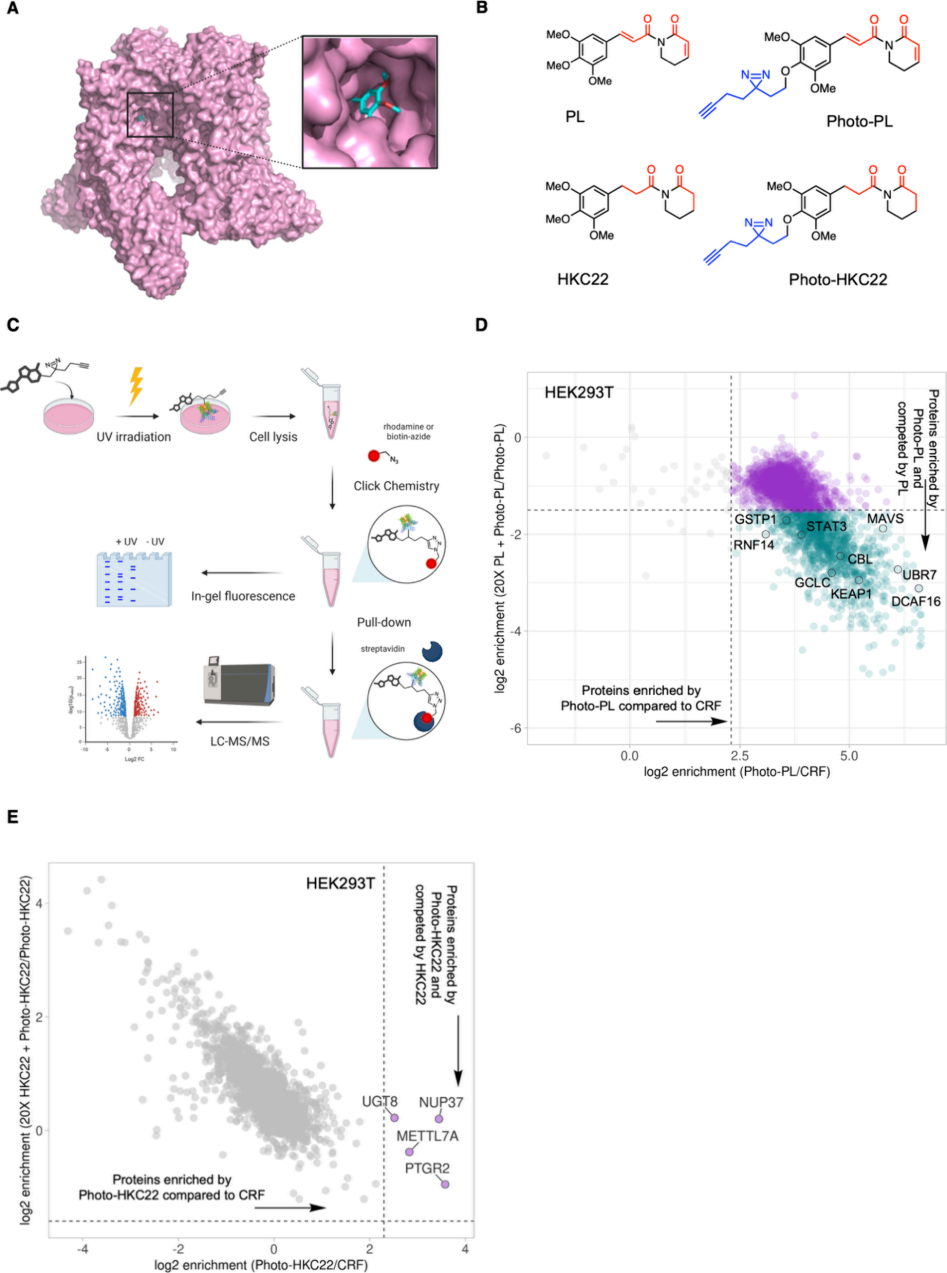
(A) Cryo-EM structure (PDB: 6WKN) of the binding mode of PL (cyan) bound in TRPV2, zoomed in on the solvent-exposed *para*-phenyl position of PL. (B) Chemical structures of PL, photoaffinity probe Photo-PL, HKC22, and photoaffinity probe Photo-HKC22. Electrophilic Michael acceptors (or lack thereof) are highlighted in red, and the minimal photo-cross-linker composed of an alkyl diazirine photoactivatable group and alkyne handle are highlighted in blue. (C) Photoaffinity labeling workflow schematic. Cells are treated with the photoaffinity probe, UV irradiated (365 nm), and lysed, and a fluorophore or biotin is conjugated to the probe via the copper-catalyzed azide–alkyne click reaction (CuAAC) for visualization of protein photolabeling by in-gel fluorescence or LC-MS/MS analysis. (D) HEK293T cells were pretreated with a 20-fold excess of PL (400 μM) or DMSO for 30 min and then cotreated with Photo-PL (20 μM) for 5 min. Cells were subsequently irradiated with UV light (365 nm) for 5 min, lysed, and conjugated with biotin-azide via CuAAC. Photolabeled proteins were enriched via pull-down with streptavidin beads and then subjected to proteomic analysis. Photo-PL and CRF (20 μM) treatments were compared. Thresholds for relative FC enrichment, represented as the log2FC, were set to 2.3 (5-fold) for enrichment of Photo-PL-labeled proteins compared to those enriched by the CRF and −1.58 (3-fold) for competition of Photo-PL-labeled proteins with excess PL; FC raw *p*-value threshold set to 0.05 (*n* = 2 for each treatment condition). (E) HEK293T cells were pretreated with a 20-fold excess of HKC22 (400 μM) or DMSO for 30 min and then cotreated with Photo-HKC22 (20 μM) for 5 min. Cells were subsequently irradiated with UV light (365 nm) for 5 min, lysed, and conjugated with biotin-azide via CuAAC. Photolabeled proteins were enriched via pull-down with streptavidin beads and then subjected to proteomic analysis. Photo-HKC22 and CRF (20 μM) treatments were compared. Thresholds for relative FC enrichment, represented as log2FC, were set to 2.3 (5-fold) for enrichment of Photo-HKC22-labeled proteins compared to those enriched by the CRF and −1.58 (3-fold) for competition of Photo-HKC22-labeled proteins with excess HKC22; FC raw *p*-value threshold set to 0.05 (*n* = 2 for each treatment condition).

Molecular docking of Photo-HKC22 in the PL-bound TRPV2 structure confirmed that the PAL linker could be accommodated without steric hindrance (Figure S6A). Consistent with this, both photoaffinity probes phenocopied the effects of PL and HKC22 on PANC-1 cell viability (Figure S6B). The overall PAL workflow for either visualization of protein photolabeling by in-gel fluorescence or detection of probe-labeled proteins by LC-MS/MS is outlined (Figure 4C).

The optimal concentration of PAL probe can vary with the system under study.^50^ Hence, we first performed initial gel fluorescence studies to determine suitable probe concentrations for subsequent competition and LC-MS/MS studies. HEK293T cells were treated with a range of concentrations of Photo-PL and Photo-HKC22, UV irradiated (365 nm, 5 min, on ice), lysed, and subjected to CuAAC conjugation with TAMRA-azide. SDS-PAGE and fluorescence imaging revealed clear UV- and concentration-dependent protein labeling for both probes (Figure S6D,F). As expected, Photo-PL exhibited partial UV-independent labeling due to its intrinsic electrophilic reactivity (Figure S6F). We performed PAL in live cells rather than in cell lysate as this preserves the native subcellular localization of targets, and the two settings have been shown to produce distinct sets of protein hits.^48,51^

From these results, we selected a probe concentration of 20 μM for subsequent experiments. To determine whether the UV-dependent bands arose from nonspecific diazirine labeling, we repeated the PAL workflow using 20 μM Photo-HKC22 or Photo-PL with pretreatment of increasing concentrations (up to 20-fold excess) of the parent compound HKC22 or PL (Figure S6E,G). Varying the concentration of the parent competitor required to compete an individual protein band has been shown to reflect its binding affinity.^52^ However, after correcting for protein loading, no significant reduction in fluorescence intensity was observed for any major bands labeled by Photo-HKC22, indicating that they likely resulted from nonspecific diazirine-mediated labeling (Figure S6E).

To achieve higher sensitivity beyond that of in-gel fluorescence, we next performed LC-MS/MS proteomic analysis of the probe-labeled proteome (Figure 4C). The same cell line (HEK293T) and treatment conditions (20 μM probe; 5 min UV irradiation) were used as in the in-gel fluorescence assays. In the resulting scatter plots (Figure 4D,E), the *x*-axis represents FC enrichment of proteins labeled by the PAL probe relative to a simple diazirine- and alkyne-containing control constant region fragment (CRF; Figure S6C), used to exclude proteins likely labeled nonspecifically by the diazirine. The *y*-axis indicates competition by pretreatment with excess parent compound (HKC22 or PL), with proteins in the lower-right quadrant (enriched and competed) considered putative “true” targets. Only four proteins were enriched by Photo-HKC22 relative to the CRF, and none showed significant competition by HKC22 (Figure 4E), indicating no identifiable direct targets under these conditions. In contrast, performing the same workflow with Photo-PL and PL revealed numerous enriched and competed proteins, including many known covalent and noncovalent targets of PL, such as GSTP1, GSTO1, KEAP1, and STAT3 (Figure 4D). This stark difference confirms the validity and sensitivity of the PAL approach while underscoring the markedly reduced off-target activity and enhanced selectivity of HKC22 relative to PL.

A key objective of these PAL experiments was to confirm direct labeling of TRPV1, TRPV2, and TRPA1 by Photo-HKC22. However, HEK293T cells express TRPA1 ^53,54^ and TRPV2 ^53,56^ at very low levels and TRPV1 ^53,55,56^ at low levels, which likely prevented their detection. HEK293T cells were nonetheless selected for initial optimization due to their high proteome coverage^57^ and the availability of a curated list of proteins frequently labeled nonspecifically by diazirines (“frequent hitters”), which were excluded from the analysis.^58^ Taken together, the absence of detectable off-targets of Photo-HKC22, in contrast with the broad labeling observed with Photo-PL, suggests that HKC22 exhibits a highly selective binding profile toward TRPV1, TRPV2, and TRPA1. Having established the selectivity and direct TRPV2 engagement of the PL derivatives, we next evaluated their functional effects in in vitro and in vivo models of cancer metastasis.

### Noncovalent PL Derivative Inhibits Cancer Cell Migration In Vitro and In Vivo

TRPV2 is emerging as an important antimetastasis target; nonselective TRPV2 antagonists have been shown to impair cancer cell migration without affecting cell viability.^16^ Having shown that the PL derivatives do not significantly affect the viability of multiple cancer cell lines (Figures 1D,E and S2), we next investigated whether they could inhibit cancer cell migration. We assessed the effect of PL and PL derivatives (HKC22 and HKC54) on cancer cell migration using in vitro wound healing assays. Confluent U-251 WT and U-251 TRPV2 knockdown (KD) cells (Figure 5D) were treated with PL, HKC22, or HKC54, and the scratch area was quantified at 0, 20, 40, and 60 h. In U-251 WT cells, all treatments significantly inhibited migration compared to that of DMSO (Figure 5A,B). In TRPV2 KD cells, HKC54 had no significant effect; HKC22 produced minimal inhibition, and PL retained activity similar to WT cells, likely reflecting its polypharmacology (Figure 5A,C). We also performed wound healing assays in confluent PANC-1 cells, treating with PL and HKC22 and quantifying scratch area at 0 and 8 h. Both compounds significantly reduced migration in TRPV2-expressing cells (Figure S7A–C). These results, consistent across two highly migratory cancer cell lines, support TRPV2 as an antimetastatic target and demonstrate that small-molecule TRPV2 antagonists can inhibit cancer cell migration in a TRPV2-dependent manner without affecting cell viability. Unlike previous studies, which exclusively examined the effects of PL derivatives on cancer cell viability, our work demonstrates their ability to inhibit cancer cell migration.^27,28,58^

**Figure 5:**
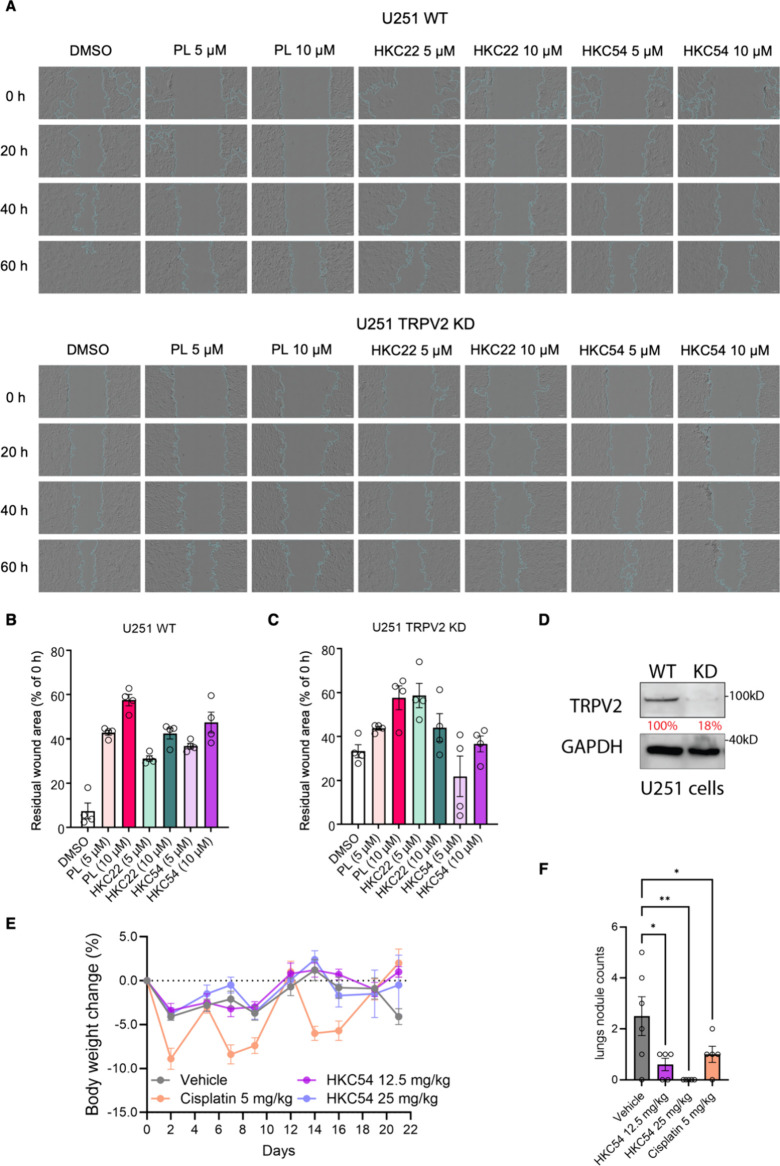
(A) Imaging of wound healing assay results in U-251 and U-251 TRPV2 KD cells after treatment with 5 or 10 μM of HKC22, HKC54, PL, or vehicle control after 20, 40, and 60 h. Images were collected by Zeiss Celldiscoverer and analyzed by ImageJ/Fiji with scratch assay plugin. The residual wound area as a percentage of the original wound area at 0 h for each treatment condition was quantified in (B) and (C) for U-251 WT and U-251 TRPV2 KD cells, respectively. (D) Immunoblot of TRPV2 protein expression in U-251 and U-251 TRPV2 KD cells. (E) Mean percent change in the body weight of Balb/c mice with breast cancer and 4T1-Luc2 lung metastasis treated with 12.5 or 25 mg/kg of HKC54, 5 mg/kg of cisplatin, or vehicle. (F) Lung nodule counts at day 21 after sacrificing the Balb/c mice with breast cancer and 4T1-Luc2 lung metastasis from each group. Data presented as mean ± SEM, ordinary one-way ANOVA test, **p < 0.01, ***p < 0.001.

Given the promising effects of the PL derivatives on in vitro cancer cell migration, we next investigated their antimetastatic activity in vivo in a 4T1-Luc2 lung metastasis model of breast cancer in Balb/c mice. This syngeneic model is widely used to study tumor growth and metastasis as it preserves the intact immune system, the host stroma, and the extracellular matrix (ECM).^59–62^ We first performed a maximum tolerated dose (MTD) study to assess the toxicity of PL derivatives in mice. Compounds were administered via intraperitoneal injection every other day for a total of five doses (Figure S8A). All derivatives were well tolerated up to 25 mg/kg, resulting in < 5% body weight loss within 5 days after the final dose (Figure S8B). Based on its superior TRPV2 selectivity in wound healing assays, we selected HKC54 for in vivo antimetastatic evaluation. Mice inoculated with 4T1-Luc2 cells were treated with HKC54 at doses of 12.5 or 25 mg/kg every other day for a total of five doses, and the body weight was monitored throughout the study. Cisplatin (5 mg/kg, twice weekly for five doses), an FDA-approved chemotherapy agent, was included as a comparator.^63,64^

HKC54 treatment did not cause body weight loss, whereas cisplatin induced a modest but tolerable decrease (Figure 5E). Notably, both HKC54 doses produced a marked reduction in lung metastatic nodules relative to cisplatin in all treated mice (Figures 5F and S8C), indicating that HKC54 effectively inhibits tumor metastasis in vivo. Overall, these findings establish HKC54 as the most potent TRPV2 antagonist reported to date and reveal its ability to inhibit cancer cell migration and metastasis without affecting the cell viability. This expands the functional landscape of PL derivatives beyond their previously reported selective cytotoxicity toward cancer cells to include selective, TRPV2-dependent antimetastatic activity.

## Discussion

In this work, we developed and characterized a novel class of selective TRPV2 antagonists derived from the natural product PL, building upon our previous machine learning-aided discovery of PL as an allosteric TRPV2 antagonist.^19^ By strategically removing the electrophilic groups of PL, we eliminated phenotypic effects associated with covalent off-target interactions, such as GSH depletion, ROS induction, and nonspecific cytotoxicity, while preserving potent TRPV2 antagonism. This rational redesign yielded HKC54, which represents the most potent TRPV2 antagonist reported to date (IC_50_ = 0.4 μM). Calcium fluorescence imaging assays in both HEK239T cells and DRG neurons, together with electrophysiological recordings in DRG neurons, confirmed the robust inhibition of TRPV2-mediated Ca^2+^ influx. Notably, HKC54 exhibited approximately 50-fold and 70-fold selectivity for TRPV2 over structurally homologous thermoTRP channels TRPV1 and TRPA1, respectively, underscoring its remarkable selectivity for TRPV2 among thermoTRP channels. Direct target engagement of TRPV2 by HKC54 and HKC22 was further supported by CETSA, while molecular docking and MD simulations suggested a near-identical binding mode of HKC54 and HKC22 to that of PL observed in our previous cryo-EM structure.^19^ Finally, HKC54 demonstrated potent antimetastatic activity in both in vitro and in vivo models of metastasis. Together, these findings establish HKC54 as a potent subtype-selective TRPV2 antagonist.

Our SAR findings align with previous studies showing that PL depletes intracellular GSH by ∼60% in EJ bladder carcinoma cells, whereas PL derivatives lacking the C2–C3 olefin fail to do so.^28^ Similarly, only compounds retaining this electrophilic site induce detectable ROS.^28^ Therefore, our results corroborate previous reports that the C2–C3 olefin, rather than C7–C8 olefin, is critical for PL’s GSH depletion, ROS induction activity, covalent reactivity, and associated cytotoxicity.^21,28^

Interestingly, our SAR analyses also revealed that subtle structural modifications to the PL scaffold confer distinct selectivity profiles across the thermoTRP channels. HKC20 emerged as a potent TRPV1 antagonist (IC_50_ = 1.7 μM), whereas HKC22 displayed inhibitory activity toward TRPV1, TRPA1, and TRPV2. TRPV1 and TRPA1 have been implicated in the pain pathway and have been the subject of extensive research for the development of novel analgesics and anti-inflammatory agents.^65^ To rationalize the additional dual antagonism of TRPA1 and TRPV1 by PL and derivatives HKC20 and HKC22, we considered their function and other known chemical modulators. Both channels sense noxious chemical stimuli, such as capsaicin, allicin, and cinnamaldehyde, and are coexpressed in many nociceptive neurons; 30–50% of TRPV1-expressing neurons also express TRPA1.^66,67^ Moreover, there is evidence that TRPV1 and TRPA1 interact to form a heterotetramer,^68–70^ and both have critical but redundant roles in acute noxious heat sensing.^71^ Correspondingly, many natural and synthetic compounds affect both channels.^72,73^ Understanding the dual antagonism was further informed by SAR studies of piperine and related black pepper-derived natural products, which act as dual TRPV1/TRPA1 agonists and are structurally similar to PL derivatives.^74,75^ In these studies, neither the degree of unsaturation nor the aliphatic chain length dictated activity (Figure S9). Instead, the piperidine ring and the methylene-dioxy phenyl substituent were the key pharmacophores.^74,75^ This is consistent with the PL derivatives, which all contain a lactam ring and trimethoxy-substituted phenyl ring and where saturated, noncovalent derivatives retain activity. Whereas piperine derivatives function as agonists, PL and its derivatives act as antagonists. Small structural modifications can switch TRP-channel modulators from agonists to antagonists; for example, demethylation of TRPA1 antagonist AP-19 converts it into an agonist.^76^ Similarly, we hypothesize that the additional carbonyl present in the lactam ring of PL and its analogues, relative to the piperine derivatives, which forms key hydrogen bonds to R539 and T522, drives this functional inversion. We also hypothesize that the α-cyano group present in HKC54 can be accommodated only in the TRPV2 binding site, which leads to its stark subtype-selectivity. These findings underscore the versatility of the PL scaffold as a foundation for designing selective or multitarget thermoTRP channel modulators. Future work to obtain structural information on the PL derivatives bound to TRPV1, TRPV2, and TRPA1 would not only validate our SAR analyses, molecular docking, and MD results but also structurally enable medicinal chemistry efforts to optimize thermoTRP channel modulators with improved pharmacokinetic properties.

We developed photoaffinity probe Photo-HKC22 based on one of our most active PL derivatives, fully saturated noncovalent HKC22. This probe enabled us to profile the proteome-wide selectivity of HKC22, revealing no detectable off-targets and suggesting high selectivity for thermoTRPs TRPV1, TRPA1, and TRPV2. In stark contrast, the photoaffinity probe derived from PL itself, Photo-PL, extensively labeled the proteome, capturing numerous known noncovalent and covalent targets of PL, such as GSTP1, GSTO1, KEAP1, and STAT3. This indicates that conjugation of the PAL linker did not perturb binding of Photo-PL, and it could be accommodated in the PL binding site of all labeled targets. This comparison highlights two important points. First, electrophilic natural products such as PL should not be assumed to engage their targets exclusively through covalency; Photo-PL captured noncovalent targets, such as GSTP1, that previous PL-derived activity-based probes failed to detect. Second, our work demonstrates that the electrophilic moieties within such natural products are not always essential for target binding; fully saturated HKC22 retained the binding and potent antagonism of TRPV2.

A limitation of our study is that we did not observe labeling of TRPV1, TRPV2, and TRPA1 by Photo-PL or Photo-HKC22, which likely reflects their very low expression in HEK239T cells. While we cannot fully exclude the possibility that the PAL linker perturbs probe binding to TRPV2, the probe design was guided by the PL-bound TRPV2 cryo-EM structure, molecular docking indicated that the PAL linker could be accommodated without steric hindrance, and the probe phenocopied the effects of HKC22 on cell viability. Moreover, Photo-PL retained the engagement of many known targets of PL, suggesting that the PAL linker does not perturb these interactions. A similar challenge was encountered in TRPC5 PAL studies and resolved by channel overexpression.^17^ Instead, we used the CETSA to demonstrate direct target engagement of TRPV2 by both HKC22 and HKC54. To further establish Photo-HKC22 as a useful ‘pocket probe’ for thermoTRPs and for future thermoTRP ligand development, subsequent studies should implement the PAL workflow either in a cell line with higher endogenous expression of thermoTRP channels or in cells engineered to overexpress TRPV2. A further limitation is that the proteome-wide selectivity of HKC54 was not evaluated, although HKC54 exhibited no phenotypic effects of irreversible covalent off-target engagement typical of PL, measured by GSH depletion, ROS induction, and cytotoxicity. Developing a photoaffinity probe analogous to Photo-HKC22 would enable the proteome-wide selectivity of HKC54 to be assessed, strengthening its use as a selective TRPV2 antagonist.

There is accumulating evidence that TRPV2 is a key driver of cancer cell migration, rather than growth.^77^ It is thought that TRPV2 enhances cancer cell migration by inducing the expression of key proteases—MMP2, MMP9, and cathepsin B—that remodel the ECM and are markers of cell invasion.^78,79^ In addition, TRPV2 maintains an elevated intracellular calcium concentration, critical to cancer cell migration ability.^79^ Our initial in vivo results in a 4T1-Luc2 lung metastasis model of breast cancer suggest that developing selective TRPV2 antagonists represents a promising strategy to combat metastasis. This is even more pertinent given that antimetastasis agents are an under-represented category of oncology drugs, in part due to multiple late-stage clinical failures and a lack of sufficient or standardized approval criteria.^80^ Moreover, although the number of deaths caused by breast cancer over the past decade is in decline, the proportion of deaths caused by metastatic disease has remained stable at 75%.^81^ Although we used an immunocompetent syngeneic murine model that is commonly used to study metastasis, we acknowledge that there is currently no single preclinical model that accurately reflects the complexity of the metastatic process in patients with cancer.^80^ Future studies with more optimized PL-based TRPV2 antagonists will involve multiple different preclinical models to better understand which step(s) in the metastatic process the compounds are inhibiting.

Finally, the development of selective, noncovalent TRPV2 antagonists brings the potential to develop targeted protein degradation (TPD) modalities. Unlike covalent inhibitors such as PL, noncovalent ligands such as HKC22 and HKC54 could serve as modular TRPV2 binders in TRPV2-targeting degraders to enable substoichiometric target turnover. TRPV2 is overexpressed in many cancers; therefore, this ability could be critical to regulating aberrant TRPV2 expression levels. For example, high TRPV2 expression characterizes the advanced and aggressive castration-resistant prostate cancer phenotype, and TRPV2 silencing was shown to reduce prostate cancer cell growth and invasion.^79^ The cryo-EM structure of PL-bound TRPV2 reveals numerous lysine residues adjacent to the PL binding site that are cytosolically accessible for ubiquitination by intracellular E3 ligases (Figure S10). Interestingly, TRIM21, a membrane-localized E3 ligase that was recently discovered to be the endogenous regulator of TRPV2 responsible for its K48-linked ubiquitination and degradation,^82^ has already been coopted for TPD using the TRIM21 ligand acepromazine in TRIM21-recruiting PROTACs (“TrimTACs”),^83^ providing a potential strategy to directly target TRPV2 for degradation. In addition, Nedd4-2 has been shown to endogenously regulate TRPA1 ^84^ and TRPV6 ^85^ and so could represent an alternative E3 ligase to target TRPV2 or TRPA1 with noncovalent PL derivatives as the protein-binding ligand. Together, these findings highlight promising opportunities for the rational development of TRPV2-directed degraders that may further potentiate the antimetastatic effects already demonstrated by HKC54.

## Supplementary Material







## Notes

Additional experimental methods, figures, and methods including compound synthesis procedures (PDF)
Competition scatter of Photo-HKC22-20x-HKC22 (XLSX)
Competition scatter of Photo-PL-20x-PL (XLSX)
