# Supplementary material for: Selective TRPV2 Antagonists Derived from the Natural Product Piperlongumine Inhibit Cancer Cell Migration and Metastasis
Source: ACS Chem Biol. 2026 Mar 25;21(4):674–88. doi: 10.1021/acschembio.5c00935 (PMC13097083; doi:10.1021/acschembio.5c00935)
Supplement: Supplementary file 1 [file cb5c00935_si_001.pdf]

SUPPORTING INFORMATION

Selective TRPV2 Antagonists Derived from the Natural Product Piperlongumine Inhibit Cancer Cell Migration and Metastasis

Hannah Kiely-Collins,[1] Cong Tang,[2] Marta C. Marques,[2] Laura Butron,[3] Angela Lamberti,[3] Nicholas Bossons,[4,5] Fabian Offensperger,[6] Vincenth Brennsteiner,[6] Bárbara Sousa,[2] Luís Carvalho,[1] Antonio Ferrer-Montiel,[3] Francisco Corzana,[7] Georg E. Winter,[6] Asia Fernandez-Carvajal,[3] and Gonçalo J. L. Bernardes[1,7]*

[1]Yusuf Hamied Department of Chemistry, University of Cambridge, Lensfield Road, Cambridge, CB2 1EW, United Kingdom.

[2]GIMM - Gulbenkian Institute for Molecular Medicine; Avenida Prof. Egas Moniz, 1649-028 Lisboa, Portugal.

[3]Instituto de Investigación, desarrollo e innovación en biotecnología sanitaria de Elche (IDiBE), Universidad Miguel Hernandez, Avda. Universidad s/n, 03202 Elche, Spain.

[4]Chemprecise Lda, 2560-247 Torres Vedras, Portugal.

[5]Research Institute for Medicines (iMed.ULisboa), Faculty of Pharmacy Universidade de Lisboa, Lisbon 1649-003, Portugal.

[6]CeMM Research Center for Molecular Medicine of the Austrian Academy of Sciences, 1090 Vienna, Austria.

[7]Departamento de Química, Universidad de La Rioja. Instituto de Investigación en Química (IQUR), 26006 Logroño, Spain.

[7]Translational Chemical Biology Group, Spanish National Cancer Research Centre 26 (CNIO), Madrid 28029, Spain.

Correspondence: gb453@cam.ac.uk

TABLE OF CONTENTS

1 Supplementary Figures 4

2 Experimental procedures 15

2.1 General chemistry experimental procedures 15

2.2 Synthetic schemes 16

2.3 Synthetic procedures 17

2.4 Cell lines and cell culture 26

2.5 GSH/GSSG-Glo™ assay 27

2.6 Fluorescence imaging of oxidative stress 27

2.7 CellTiter-Glo® luminescent cell viability assay 28

2.8 Fluorescence imaging of intracellular calcium in HEK293T cells 28

2.9 Primary cultures of rat DRG nociceptors 29

2.10 Fluorescence imaging of intracellular calcium in DRG nociceptors 29

2.11 Electrophysiological recordings with DRG nociceptors 30

2.12 Photoaffinity labelling methods 31

2.12.1 Photoaffinity labelling probe concentration determination for in-gel fluorescence 31

2.12.2 Photoaffinity labelling competition study for in-gel fluorescence 31

2.12.3 In-situ labelling of cells with photoaffinity probe for proteomics 32

2.12.4 Preparation of probe-labeled proteome for MS-based protein analysis 32

2.12.5 2D-RP/RP Liquid Chromatography – Tandem Mass Spectrometry analysis 34

2.12.6 Peptide and protein identification and quantification 35

2.13 Cellular thermal shift assay (CETSA) 37

2.14 Wound healing assay in PANC-1 cells 37

2.15 Wound healing assay in U-251 and U-251 TRPV2 KD cells 37

2.16 Computational methods 38

2.16.1 Protein visualisation and figure creation software 38

2.16.2 Molecular docking of piperlongumine and derivatives 38

2.16.3 Molecular dynamics simulations of HKC22 bound to TRPV2 38

2.17 Maximum tolerated dose (MTD) test 39

2.18 In vivo metastasis model 39

1 Supplementary Figures

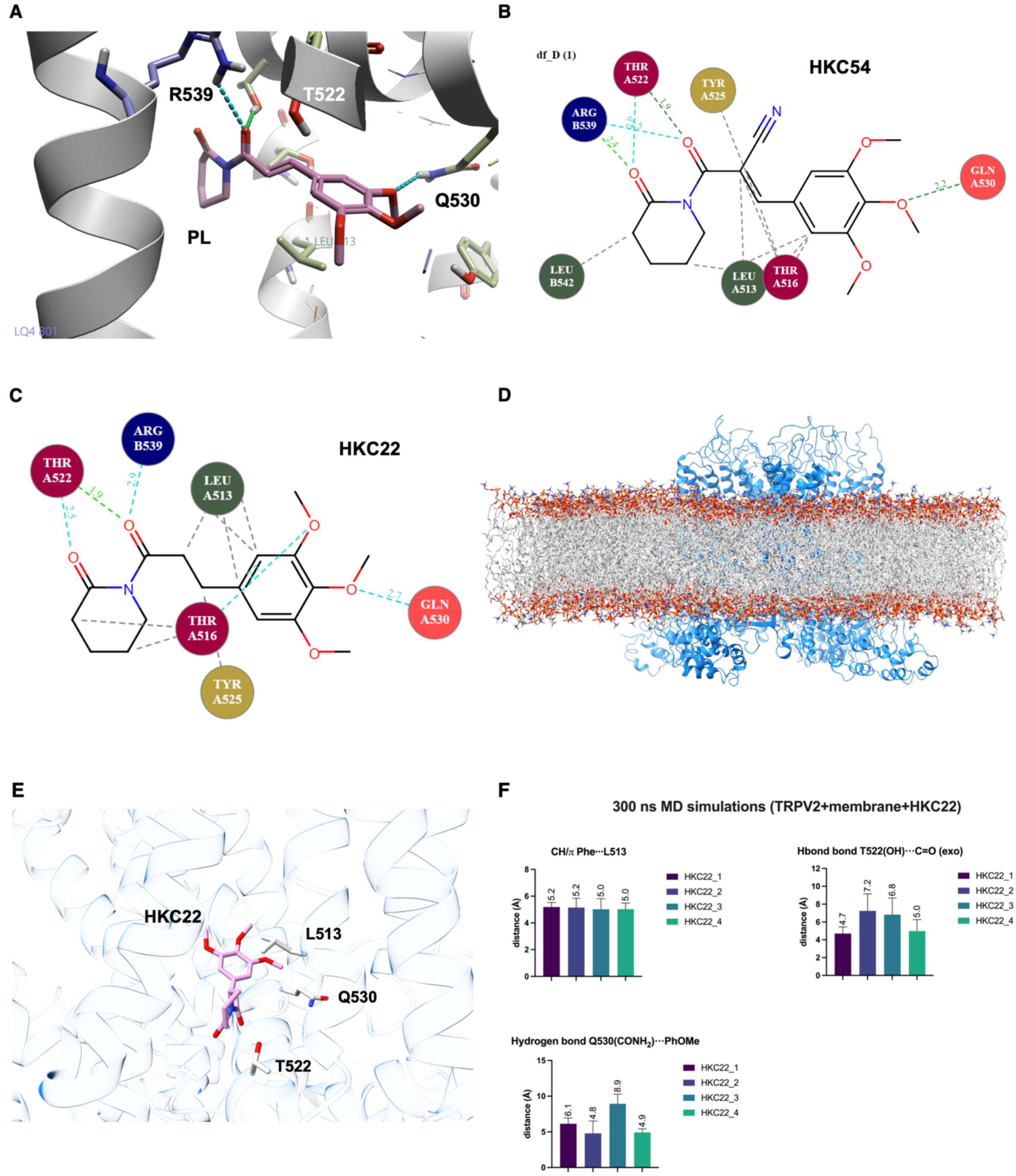

FIGURE S1 I A) Hydrogen bonds formed by PL to TRPV2 (PDB: 6WKN). Hydrogen bonding interactions displayed as green and blue dotted lines. **B**), **C**) 2D interaction maps of the key interactions formed to key residues on TRPV2 by HKC54 and HKC22, respectively, after molecular docking into PL-bound TRPV2 structure (PDB: 6WKN). Hydrogen bonding interactions displayed as green and blue dotted lines and hydrophobic interactions as grey dotted lines. **D**) Representative frame derived from a 300 ns MD simulation of HKC22 bound to TRPV2, with the complex embedded in a mimetic membrane. The protein is depicted as

blue cartoons, and the lipids are shown as stick representations. **E**) Close-up view of the representative frame, highlighting HKC22 (purple) along with the key interacting residues L513, Q530 and T522 of TRPV2. The protein is depicted as white ribbons. **F**) Distances between the ligand and key interacting residues L513, Q530 and T522 of the protein across the four subunits of the TRPV2 complex. Data are presented as mean ± SD.

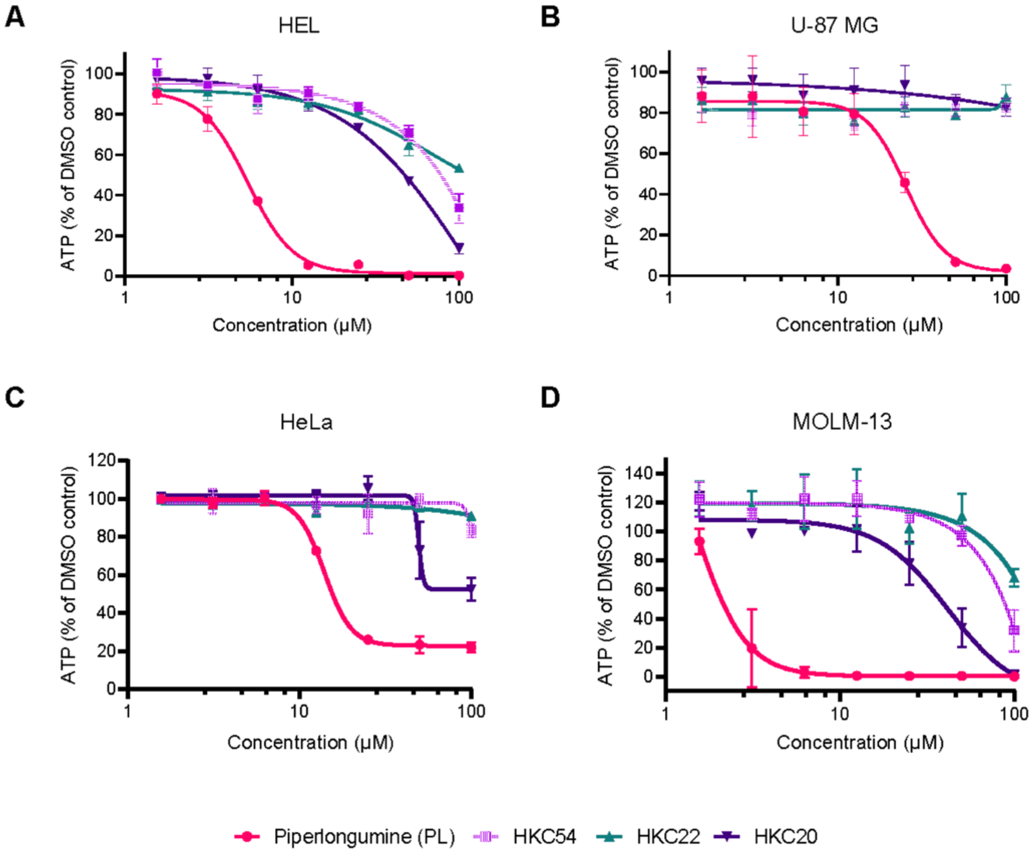

	IC$_{50}$ values (µM)<br>pIC$_{50}$ ± SD			
	PL	HKC54	HKC20	HKC22
U-251	26.6<br>4.6 ± 0.1	-	-	-
U-251<br>TRPV2 KD	31.0<br>4.5 ± 0.1	-	-	-
U-87 MG	25.4<br>4.6 ± 0.1	-	-	-
HEL	5.0<br>5.3 ± 0.1	74<br>4.1 ± 0.1	43<br>4.4 ± 0.1	-
MOLM-13	1.5<br>5.8 ± 0.1	-	41<br>4.4 ± 0.2	-
HeLa	14.0<br>4.9 ± 0.03	-	49<br>4.3 ± 0.1	-

SD = standard deviation
Time = 48 h

FIGURE S2 I A), B), C) and **D)** Cell viability results of PL, HKC54, HKC22 and HKC20 treatment after 48 h, measured using CellTiter-Glo® in HEL, U-87 MG, HeLa and MOLM-13 cell lines, respectively. Data are shown as mean ± SD (n = 3).

TRPV1

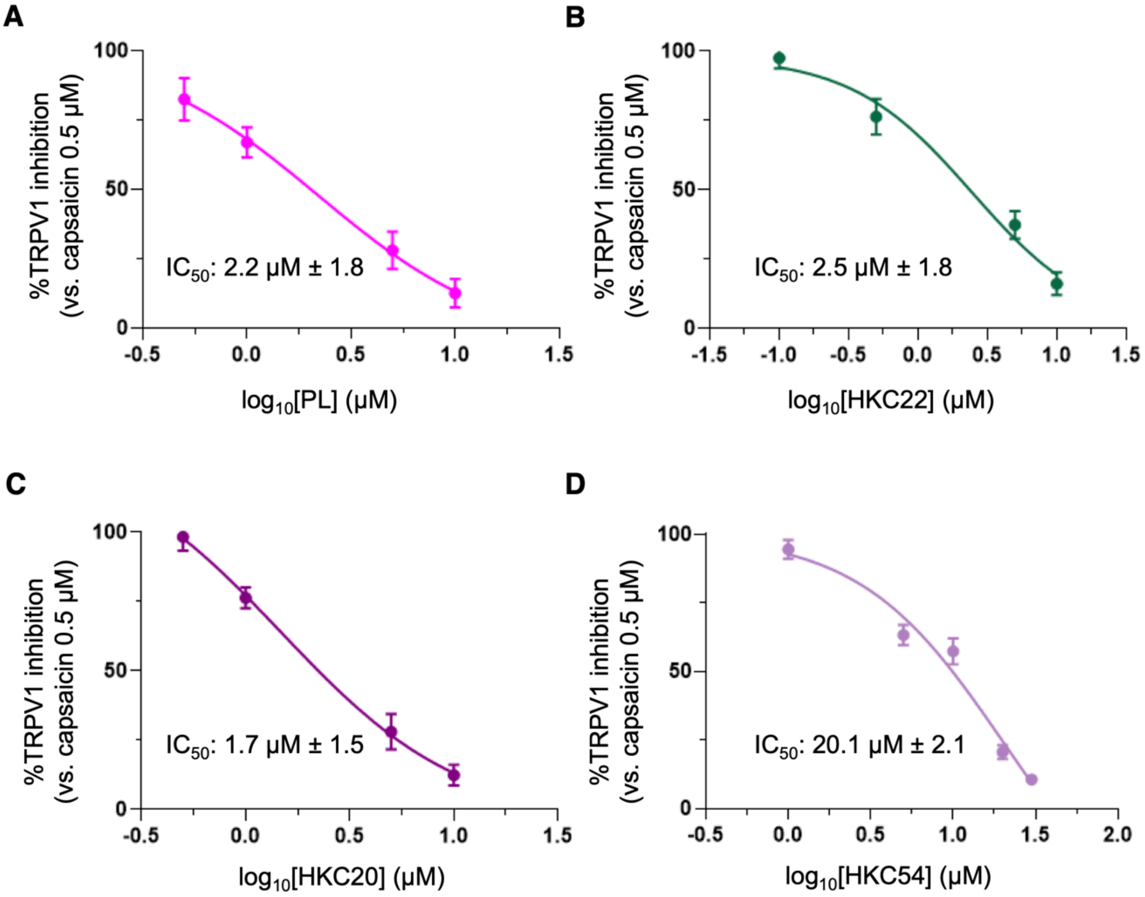

FIGURE S3 I A), **B)**, **C)** and **D)**, Dose-response % inhibition of TRPV1 upon compound treatment with PL, HKC22, HKC20 and HKC54, respectively, following TRPV1 agonism with capsaicin (0.5 µM).

TRPA1

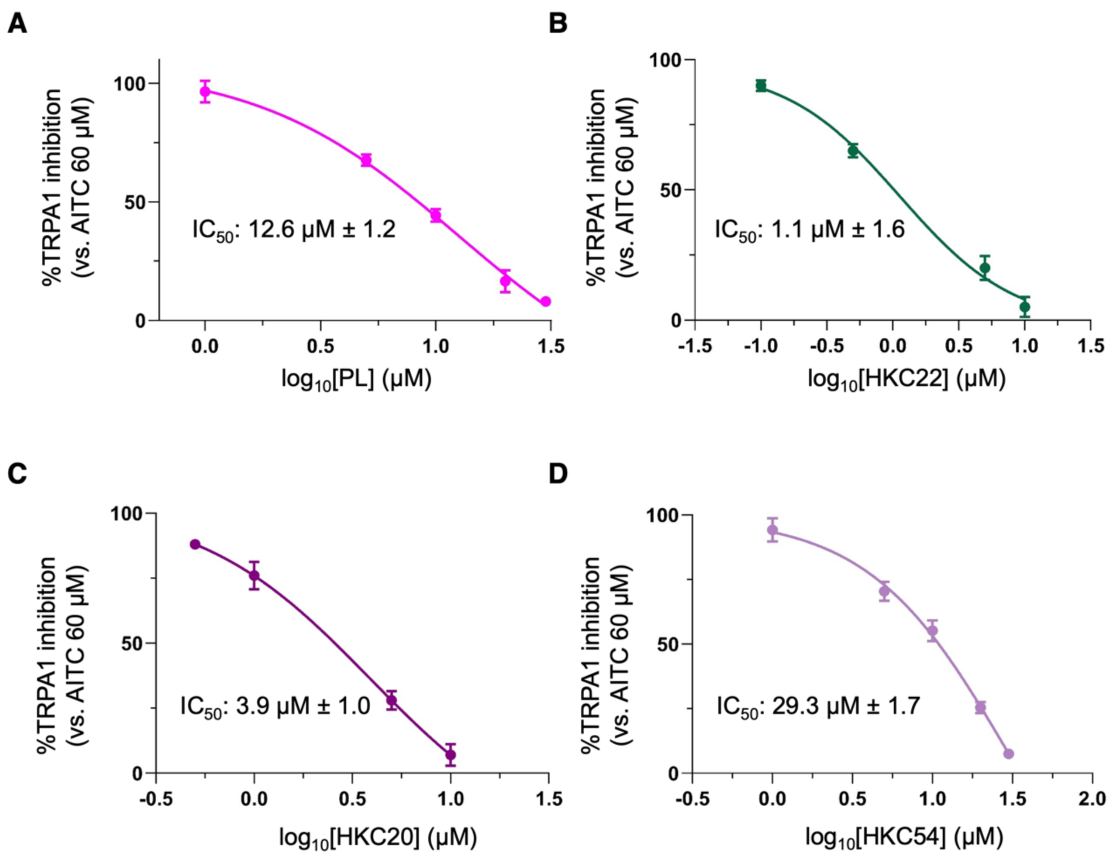

FIGURE S4 I A), **B)**, **C)** and **D),** Dose-response % inhibition of TRPA1 upon compound treatment with PL, HKC22, HKC20 and HKC54, respectively, following TRPA1 agonism with AITC (60 μM).

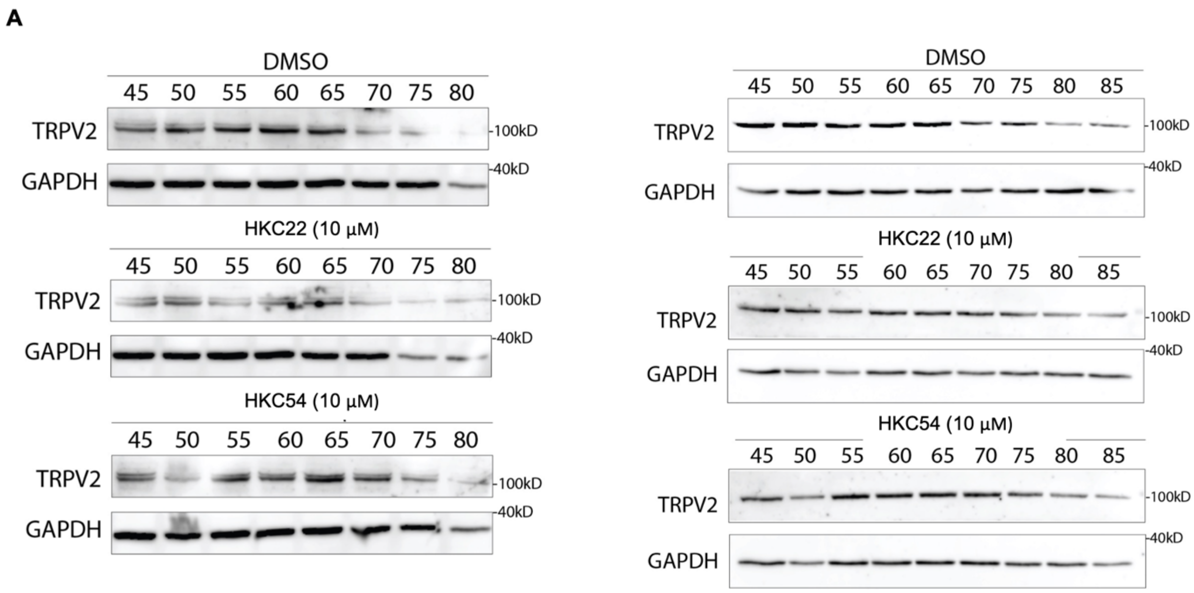

FIGURE S5 I A) Cellular thermal shift assay (CETSA) immunoblot results from repeats 2 and 3.

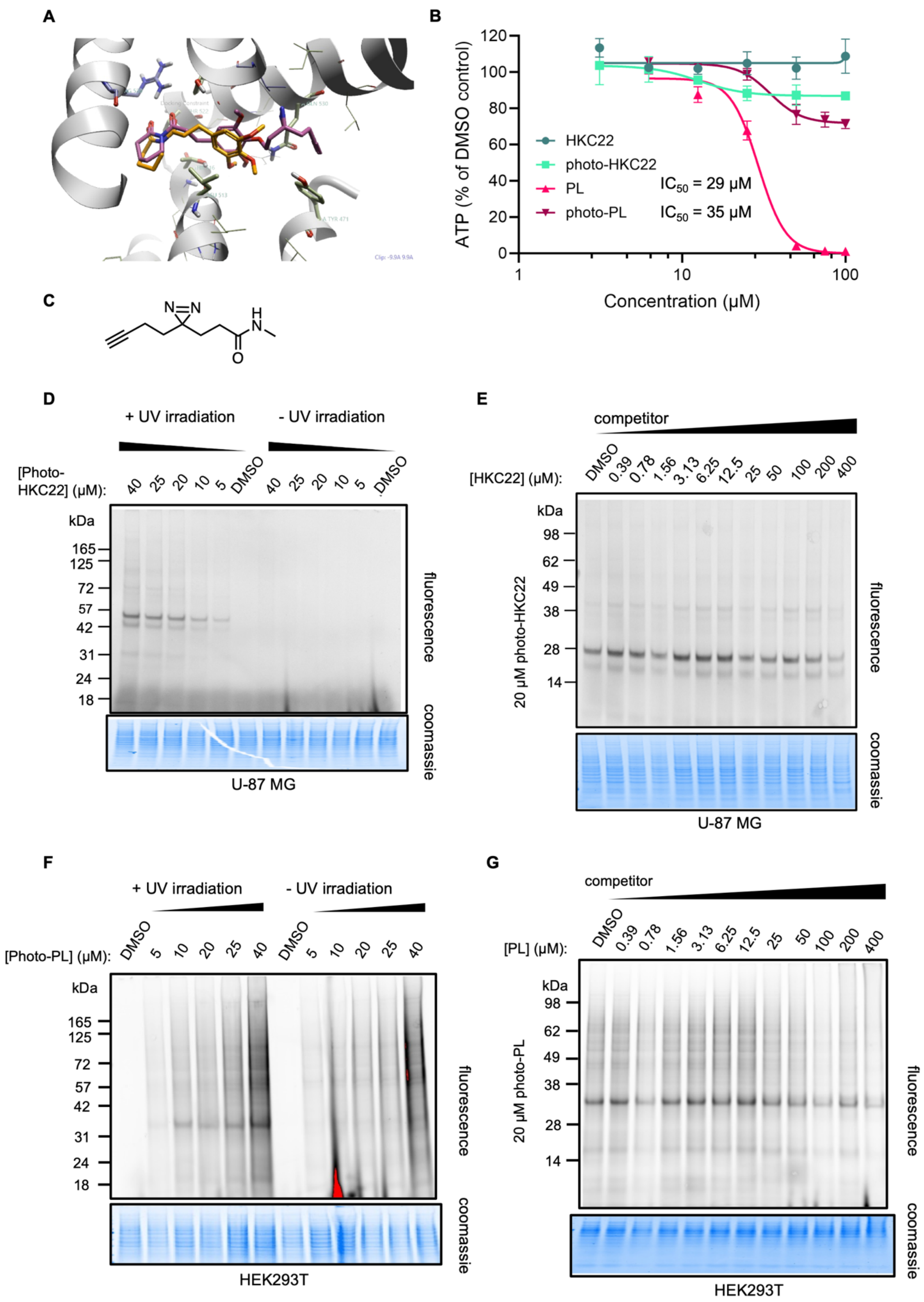

FIGURE S6 I A) Molecular docking of HKC22 (orange) overlaid with Photo-HKC22 (pink) in the PL-bound TRPV2 structure (PDB: 6WKN) showing that the minimal diazirine-alkyne photocrosslinker can be accommodated in the PL binding site and Photo-HKC22 can adopt

the same binding pose as HKC22. **B**) Cell viability results measured using CellTiter-Glo® of PL, HKC22 and Photo-HKC22 treatment in PANC-1 cells after 48 h. **C**) Chemical structure of the constant region fragment (CRF) used in the photoaffinity labelling proteomics experiments. **D**) U-87 MG cells were treated with Photo-HKC22 (5, 10, 20, 25, 40 µM) or DMSO vehicle for 1 h. Cells were subsequently UV irradiated (365 nm) for 5 min on ice, lysed and conjugated with rhodamine-azide via CuAAC. Proteins were resolved by SDS-PAGE and visualised by in-gel fluorescence. Protein loading was assessed by Coomassie staining. **E)** U-87 MG cells were pre-treated with HKC22 (400, 200, 100, 50, 25, 12.5, 6.25, 3.13, 1.56, 0.78, 0.39 µM) or vehicle DMSO for 45 min, then co-treated with Photo-HKC22 (20 µM) for 1 h. Cells were subsequently UV irradiated (365 nm) for 5 min on ice, lysed and conjugated with rhodamine-azide via CuAAC. Proteins were resolved by SDS-PAGE and visualised by in-gel fluorescence. Protein loading was assessed by Coomassie staining. **F)** HEK293T cells were treated with Photo-PL (5, 10, 20, 25, 40 µM) or DMSO vehicle for 1 h. Cells were subsequently UV irradiated (365 nm) for 5 min on ice, lysed and conjugated with rhodamine-azide via CuAAC. Proteins were resolved by SDS-PAGE and visualised by in-gel fluorescence. Protein loading was assessed by Coomassie staining. **G)** HEK293T cells were pre-treated with PL (400, 200, 100, 50, 25, 12.5, 6.25, 3.13, 1.56, 0.78, 0.39 µM) or vehicle DMSO for 45 min, then co-treated with Photo-PL (20 µM) for 1 h. Cells were subsequently UV irradiated (365 nm) for 5 min on ice, lysed and conjugated with rhodamine-azide via CuAAC. Proteins were resolved by SDS-PAGE and visualised by in-gel fluorescence. Protein loading was assessed by Coomassie staining.

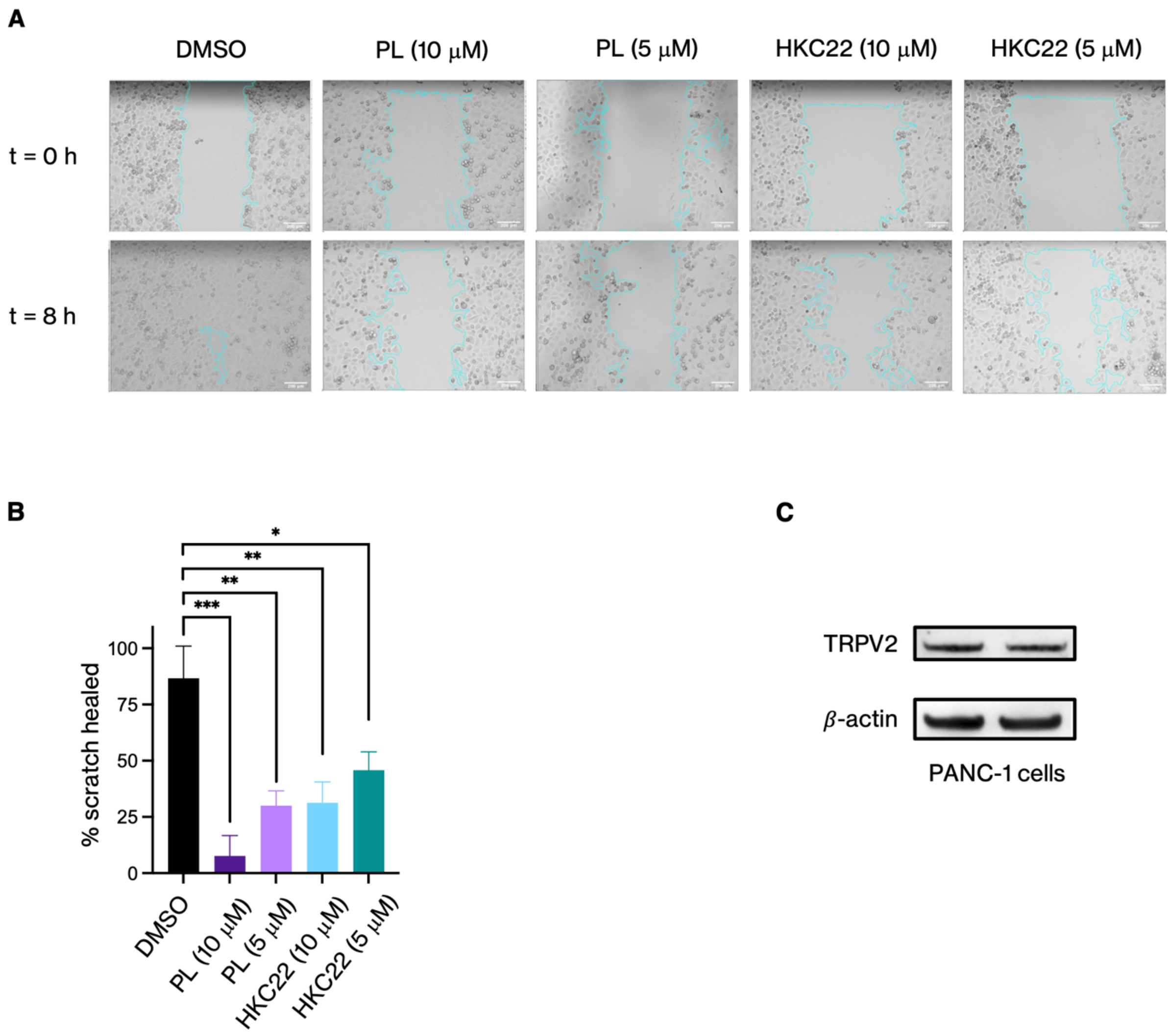

FIGURE S7 I A) wound healing assay in PANC-1 cells after treatment with vehicle control, PL or HKC22. Scratch area measured after 8 h using ImageJ. **B)** Wound healing assay results quantified. Statistical significance was calculated with one-way ANOVA and Tukey's multiple comparisons test. *$p < 0.05$, **$p < 0.01$, ***$p < 0.001$. **C)** Immunoblot of TRPV2 in PANC-1 (n = 2) with β-actin as loading control.

A

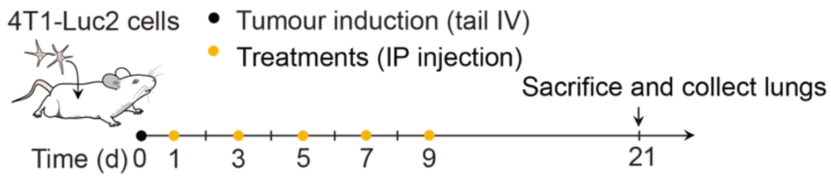

B

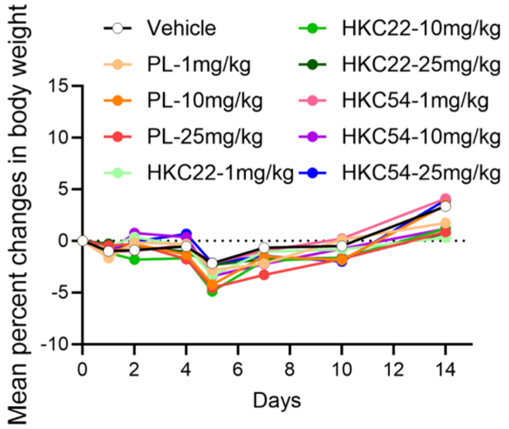

C

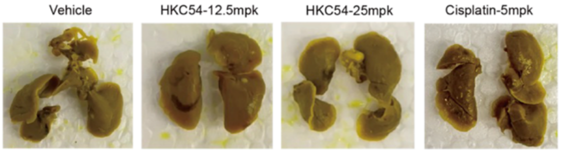

FIGURE S8 I A) *in vivo* study scheme with Balb/c mice. Intravenous tumour induction with 4T1-Luc2 cells on day 0 followed by treatment with HKC54 (12.5 or 25 mg/kg) on alternate days and sacrifice and collection of the lungs after 21 days. **B)** Maximum tolerated dose (MTD) study of PL, HKC22 and HKC54 after 14 days. **C)** Representative images of the lung nodule counts in the vehicle, cisplatin or HKC54-treated mice after 21 days.

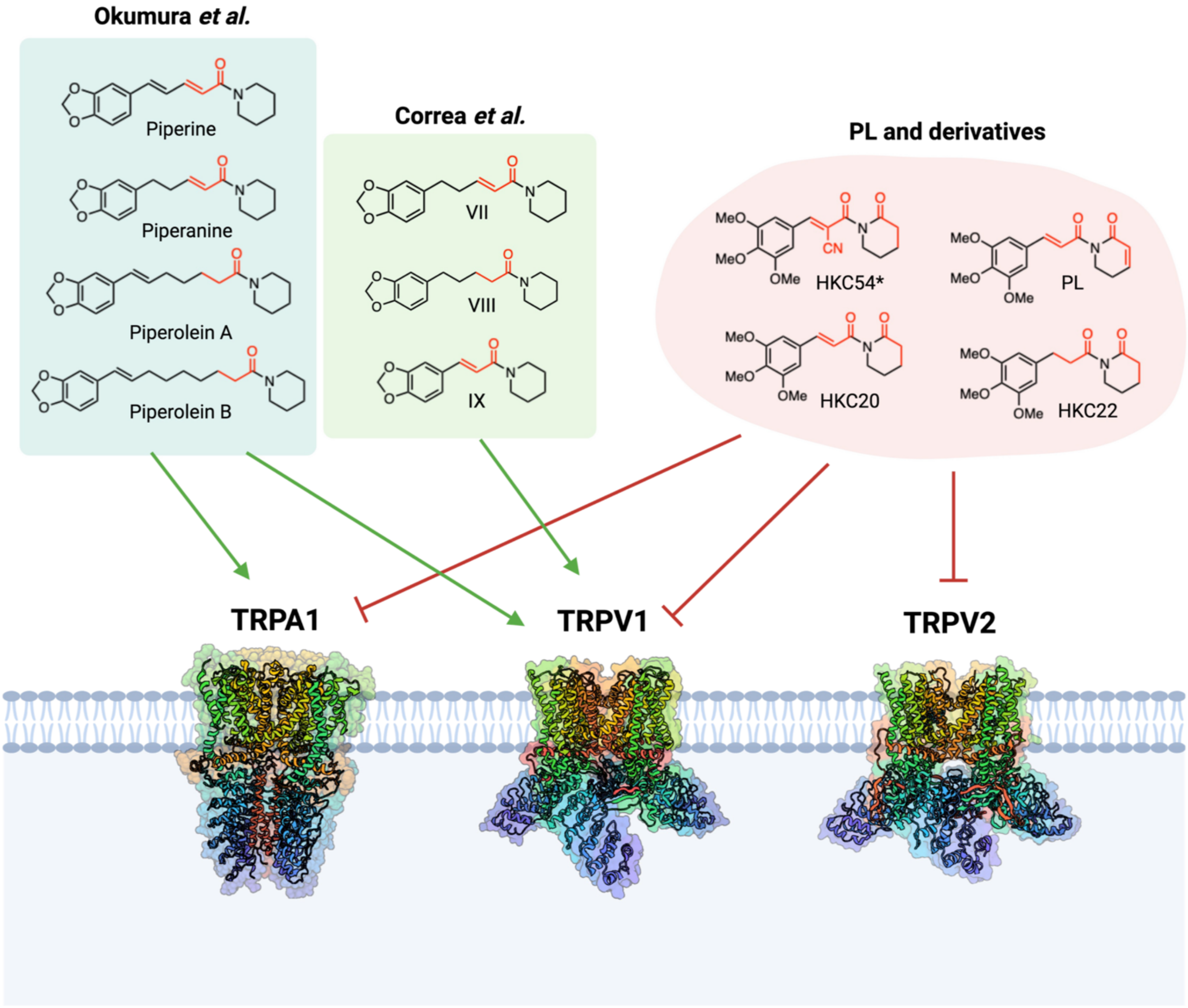

FIGURE S9 I Chemical structures of piperine derivatives known to act as dual TRPV1 and TRPA1 agonists. Electrophilic Michael acceptors (or lack thereof) highlighted in red. *HKC54 is a selective TRPV2 antagonist.

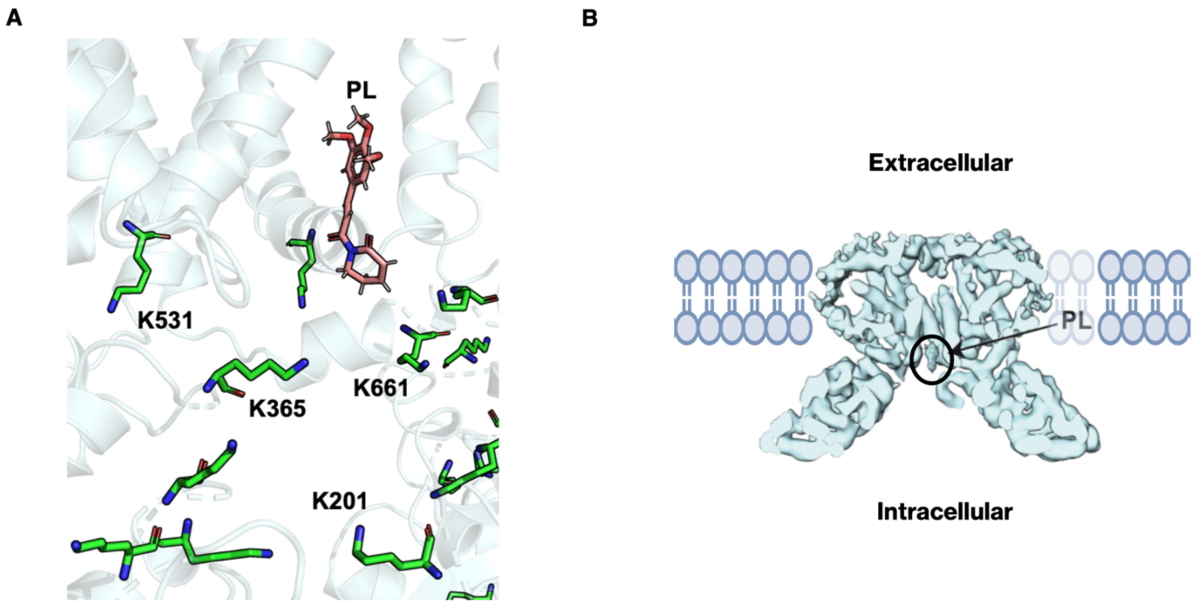

FIGURE S10 I A) Lysine residues adjacent to the PL binding site in TRPV2 (PDB: 6WKN). **B)** PL binding site is intracellularly-accessible from the cryo-EM structure of PL-bound TRPV2 (PDB: 6WKN).

2 Experimental procedures

2.1 General chemistry experimental procedures

All non-aqueous reactions were performed in oven-dried glassware under a dry argon or nitrogen atmosphere with dry solvents unless otherwise stated. Reaction vessels were heated using thermostatically controlled DrySyn blocks with the liquid level of the flask below that of the heating block. Reaction temperatures refer to the thermostat set-point. A reaction temperature of 0 °C refers to an external ice/water slurry cooling bath. A reaction temperature of -20 °C refers to an external dry ice/ethylene glycol slurry cooling bath. A reaction temperature of -78 °C refers to an external dry ice/acetone slurry cooling bath. All reagents were purchased from commercial sources and used without further purification unless otherwise stated. CH_2Cl_2, THF and Et_2O were purified either according to the method of Grubbs and Pangborn[1] or by distillation under an inert atmosphere (CH_2Cl_2, MeOH and MeCN were distilled from calcium hydride. THF and Et_2O were pre-dried over sodium wire then distilled from calcium hydride and lithium aluminium hydride). Petroleum ether, *n*-hexane and EtOAc were distilled on site. 'Petrol' refers to the distillate of petroleum ether collected between 40-60 °C unless otherwise stated. Water used experimentally was deionised and prepared on site.

Flash column chromatography was performed using Merck silica gel 60 Å (40 - 63 µm). Analytical thin layer chromatography was performed using Merck Silica gel 60 F_{254} 1 mm glass

plates and visualised by UV (254 nm) or by staining with an indicated solution prepared by known procedures. Preparative thin layer chromatography was performed using Merck Silica gel 60 F_{254} 2 mm glass plates and visualised by UV (254 nm).

NMR spectra were recorded on 400 MHz Avance III HD, 400 MHz Neo 400 and 600 MHz Avance 600 BBI spectrometers. Chemical shifts are reported in parts per million (ppm) and the spectra are calibrated to the residual solvent peak (^{1}H NMR: $CDCl_3$ δ 7.26 ppm; ^{13}C NMR: $CDCl_3$ δ 77.16 ppm). Multiplicities are described as s (singlet), d (doublet), t (triplet), q (quartet), m (multiplet), dd (double doublet) etc. Coupling constants (*J*) are reported in hertz (Hz) to 1 decimal place using Mestrenova software version 12.0.0 for signal processing. The centre of each peak is reported except for multiplet signals where a range of ppm values are given. High-resolution mass spectra (HRMS) were obtained with a Waters Xevo G2-S TOF mass spectrometer.

2.2 Synthetic schemes

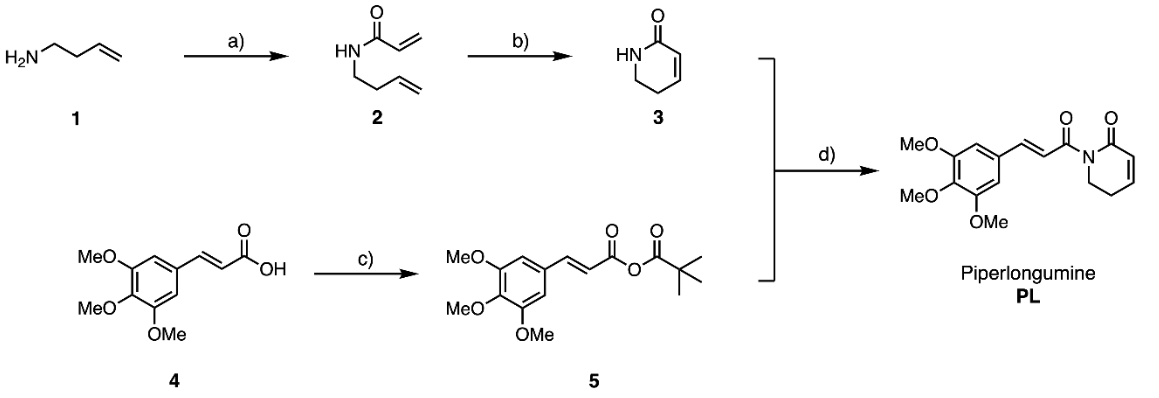

Scheme 1. Synthesis of piperlongumine (PL).

Reagents and conditions: a) $CH_2CHCOCl$, NEt_3, CH_2Cl_2, 0 °C to rt, 3 h, 63%; b) Grubbs catalyst (2nd generation), CH_2Cl_2, reflux, 6 h, 55%; c) pivaloyl chloride, NEt_3, THF, -20 °C, 45 min; d) *n*-BuLi, THF, -78 °C, 45 min; anhydride addition, 1 h, 62%.

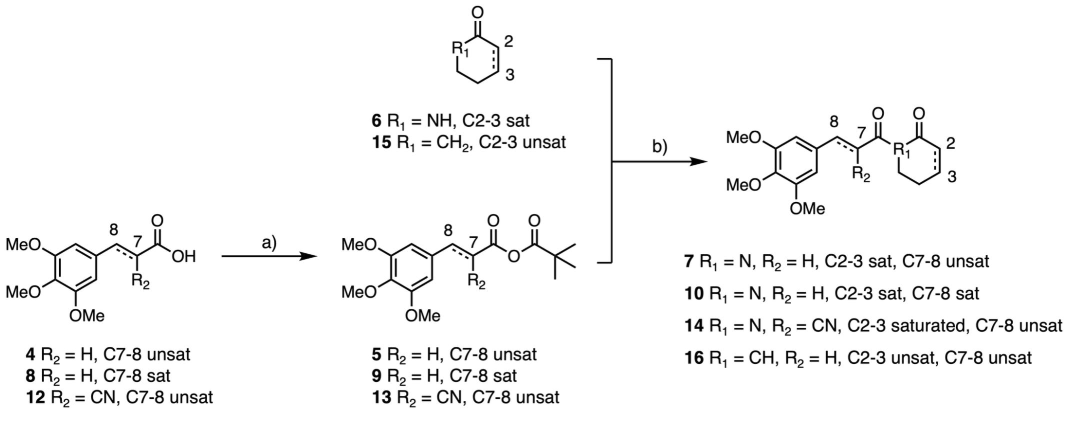

Scheme 2. Synthesis of piperlongumine derivatives HKC20 (7), HKC22 (10), HKC54 (14) and PL-0N (16).

Reagents and conditions: a) pivaloyl chloride, NEt_3, THF, -20 °C, 45 min; b) *n*-BuLi, THF, -78 °C, 45 min; anhydride addition, 1 h, 7: 85%; 10: 62%; 14: 58%; 16: 63%. Sat and unsat are saturated and unsaturated, respectively.

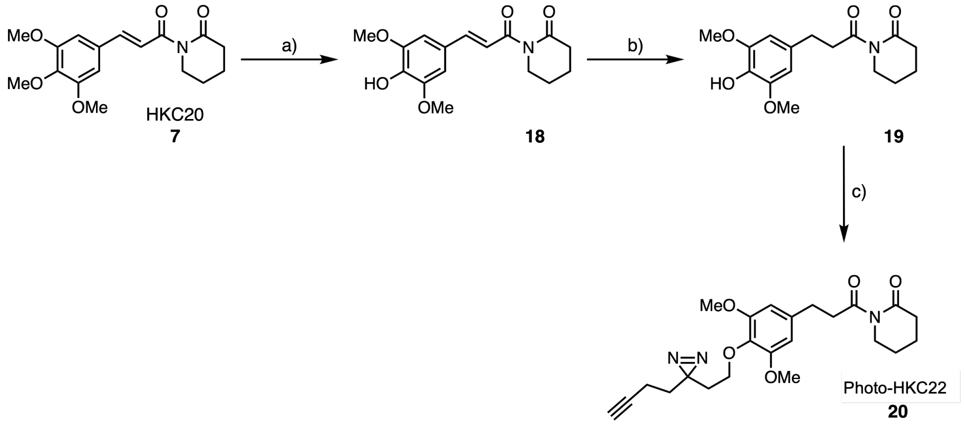

Scheme 3. Synthesis of photoaffinity probe Photo-HKC22 (20).

Reagents and conditions: a) $AlCl_3$, CH_2Cl_2, 0 °C to rt, 1.5 h, 50%; b) AcOH, $NaBH_4$, Pd/C, CH_2Cl_2, rt, 1 h, 30%; c) PAL linker **17**, tBuOK, DMF, 0 °C to rt, 18 h, 23%.

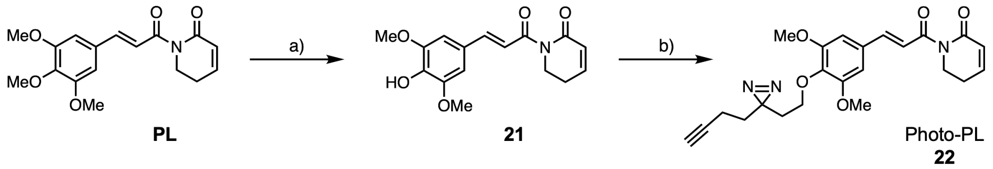

Scheme 4. Synthesis of photoaffinity probe Photo-PL (22).

Reagents and conditions: a) $AlCl_3$, CH_2Cl_2, 0 °C to rt, 1.5 h, 78%; b) PAL linker **17**, tBuOK, DMF, 0 °C to rt, 18 h, 10%.

2.3 Synthetic procedures

N-(but-3-en-1-yl)acrylamide (2)

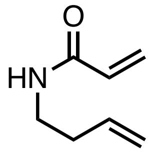

Triethylamine (2.94 mL, 21.1 mmol, 1.5 equiv.) was added to a stirred solution of but-3-en-1-amine (1.00 g, 14.1 mmol, 1.0 equiv.) in CH_2Cl_2 (20 mL) at 0 °C. Acryloyl chloride (1.37 mL, 16.9 mmol, 1.2 equiv.) was added and the mixture was stirred at room temperature for 3 h. The reaction mixture was diluted with water, and then extracted into CH_2Cl_2 (2 × 10 mL). The solvent was removed *in vacuo* and the crude product was purified by flash column chromatography on silica gel (98:2 CH_2Cl_2/MeOH) to yield **2** as a yellow oil (1.10 g, 14.1 mmol, 63%).

R_f 0.3 (98:2 CH_2Cl_2/MeOH)

^{1}H NMR (500 MHz, $CDCl_3$) δ 6.27 (ddd, *J* = 17.0, 1.5, 0.6 Hz, 1H), 6.07 (ddd, *J* = 17.0, 10.3, 0.6 Hz, 1H), 5.78 (ddt, *J* = 17.1, 10.2, 6.8 Hz, 1H), 5.63 (dt, *J* = 10.3, 1.1 Hz, 1H), 5.16 – 5.06 (m, 2H), 3.42 (tdd, *J* = 6.7, 5.7, 0.9 Hz, 2H), 2.30 (qt, *J* = 6.7, 1.3 Hz, 2H).
^{13}C NMR (126 MHz, $CDCl_3$) δ 165.6, 135.3, 131.0, 126.5, 117.5, 38.6, 33.8.
HRMS *m/z* (ESI+) $C_7H_{11}NO$ ([M+H]+) calculated 125.0841, found 125.0845
The spectroscopic data are in good agreement with literature values.[2]

5,6-dihydropyridin-2(1*H*)-one (3)

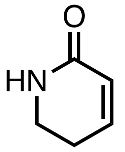

Grubbs-II catalyst (21 mg, 5 mol %) was added to a solution of *N*-(but-3-en-1-yl)acrylamide (0.0625 g, 0.5 mmol) in CH_2Cl_2 (80 mL) and refluxed for 6 h under inert conditions. The mixture was stirred for an additional 1 h at room temperature in open air to deactivate the catalyst. The reaction mixture was filtered through celite, concentrated, and the residue was purified by flash column chromatography on silica gel (1:4 hexane/EtOAc flushed with MeOH/CH_2Cl_2) to give **3** as a brown oil (26.62 mg, 0.27 mmol, 55%).
R_f 0.10 (1:4 Petrol/EtOAc)
^{1}H NMR (400 MHz, $CDCl_3$) δ 6.63 (dt, *J* = 10.0, 4.2 Hz, 1H), 6.40 – 6.27 (m, 1H), 5.88 (d, *J* = 9.9 Hz, 1H), 3.48 – 3.37 (m, 2H), 2.33 (tdd, *J* = 6.9, 4.2, 1.8 Hz, 2H).
^{13}C NMR (101 MHz, $CDCl_3$) δ 166.6, 141.7, 124.8, 39.7, 23.9.
The spectroscopic data are in good agreement with literature values.[2]

(E)-1-(3-(3,4,5-trimethoxyphenyl)acryloyl)-5,6-dihydropyridin-2(1H)-one (PL)

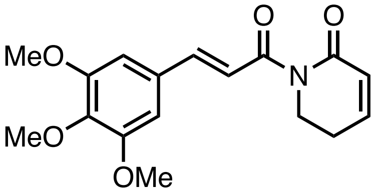

To a solution of (*E*)-3-(3,4,5-trimethoxyphenyl)acrylic acid (104.25 mg, 0.44 mmol, 1 equiv.), in freshly distilled THF (2.5 mL) was added triethylamine (0.05 mL, 0.36 mmol, 0.82 equiv.). Pivaloyl chloride (41.6 mg, 0.35 mmol, 0.79 equiv.) was added at –20 °C and the reaction mixture was stirred for 45 min. To a separate solution of **2** (50.9 mg, 0.525 mmol, 1 equiv.) in freshly distilled THF (2.5 mL) was added *n*-BuLi (0.4 mL, 0.63 mmol, 1.2 equiv.) at –78 °C under argon and the reaction was stirred for 45 min. Then, anhydride prepared from the above step was added and the reaction mixture was stirred for 1 h. The reaction mixture was quenched with saturated NH_4Cl (1 mL), extracted with ethyl acetate (2 × 5 mL), the organic layer was separated and washed with sat. NaCl (2 × 3 mL) and dried over anhydrous Na_2SO_4. The residue was evaporated *in vacuo* to give a crude product which was purified by flash

column chromatography on silica gel (3:2 Petrol/EtOAc) to yield **PL** as an off-white powder (86.6 mg, 0.27 mmol, 62%).

R*f* 0.19 (3:2 Petrol/EtOAc).

^{1}H NMR (400 MHz, $CDCl_3$) δ 7.67 (d, *J* = 15.5 Hz, 1H), 7.42 (d, *J* = 15.5 Hz, 1H), 6.94 (dt, *J* = 9.7, 4.2 Hz, 1H), 6.80 (s, 2H), 6.04 (dt, *J* = 9.7, 1.8 Hz, 1H), 4.03 (t, *J* = 6.5 Hz, 2H), 3.88 (s, 6H), 3.87 (s, 3H), 2.47 (tdd, *J* = 6.3, 4.2, 1.9 Hz, 2H).

^{13}C NMR (101 MHz, $CDCl_3$) δ 169.0, 166.0, 153.5, 145.7, 143.9, 140.1, 130.8, 125.9, 121.2, 105.6, 61.1, 56.3, 41.8, 24.9.

HRMS *m/z* (ESI+) $C_{17}H_{19}NO_5$ ([M+H]+) calculated 317.1263, found 317.1261

The spectroscopic data are in good agreement with literature values.[3]

(E)-1-(3-(3,4,5-trimethoxyphenyl)acryloyl)piperidin-2-one HKC20 (7)

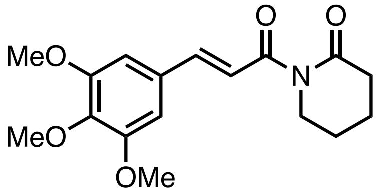

To a solution of (*E*)-3-(3,4,5-trimethoxyphenyl)acrylic acid (625.5 mg, 2.63 mmol, 1 equiv.), in freshly distilled THF (15 mL) was added triethylamine (0.26 mL, 2.16 mmol, 0.82 equiv.). Pivaloyl chloride (255 μL, 2.07 mmol, 0.79 equiv.) was added at –20 °C and the reaction mixture was stirred for 45 min. To a separate solution of piperidin-2-one (312.0 mg, 3.15 mmol, 1 equiv.) in freshly distilled THF (15 mL) was added *n*-BuLi (2.4 mL, 3.78 mmol, 1.2 equiv.) at –78 °C under argon and the reaction was stirred for 45 min. Then, anhydride prepared from the above step was added and the reaction mixture was stirred for 1 h. The reaction mixture was quenched with saturated NH_4Cl (6 mL), extracted with ethyl acetate (2 × 30 mL), the organic layer was separated and washed with sat. NaCl (2 × 18 mL) and dried over anhydrous Na_2SO_4. The residue was evaporated *in vacuo* to give a crude product which was purified by flash column chromatography on silica gel (3:2 Petrol/EtOAc) to yield **7** as an off-white powder (712 mg, 0.27 mmol, 85%).

R*f* 0.23 (3:2 Petrol/EtOAc).

^{1}H NMR (600 MHz, $CDCl_3$) δ 7.62 (d, *J* = 15.5 Hz, 1H), 7.35 (d, *J* = 15.5 Hz, 1H), 6.78 (s, 2H), 3.87 (s, 6H), 3.86 (s, 3H), 3.80 – 3.77 (m, 2H), 2.63 – 2.57 (m, 2H), 1.87 (p, *J* = 3.3 Hz, 4H).

^{13}C NMR (126 MHz, Acetone) δ 173.9, 169.7, 154.3, 142.4, 140.8, 131.5, 122.7, 106.2, 60.4, 56.2, 44.9, 35.1, 23.0, 21.1.

The spectroscopic data are in good agreement with literature values.[3]

1-(3-(3,4,5-trimethoxyphenyl)propanoyl)piperidin-2-one HKC22 (10)

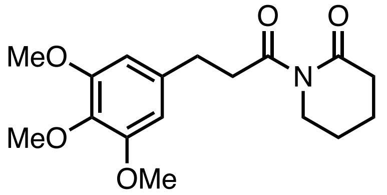

To a solution of (3-(3,4,5-trimethoxyphenyl)propanoic acid (630.7 mg, 2.63 mmol, 1 equiv.), in freshly distilled THF (15 mL) was added triethylamine (0.26 mL, 2.16 mmol, 0.82 equiv.). Pivaloyl chloride (255 μL, 2.07 mmol, 0.79 equiv.) was added at –20 °C and the reaction mixture was stirred for 45 min. To a separate solution of piperidin-2-one (312.0 mg, 3.15 mmol, 1 equiv.) in freshly distilled THF (15 mL) was added *n*-BuLi (2.4 mL, 3.78 mmol, 1.2 equiv.) at –78 °C under argon and the reaction was stirred for 45 min. Then, anhydride prepared from the above step was added and the reaction mixture was stirred for 1 h. The reaction mixture was quenched with saturated NH_4Cl (6 mL), extracted with ethyl acetate (2 × 30 mL), the organic layer was separated and washed with sat. NaCl (2 × 18 mL) and dried over anhydrous Na_2SO_4. The residue was evaporated *in vacuo* to give a crude product which was purified by flash column chromatography on silica gel (3:2 Petrol/EtOAc) to yield **10** as a white powder (524.03 mg, 1.63 mmol, 62%).

R_f 0.19 (3:2 Petrol/EtOAc).

^{1}H NMR (600 MHz, $CDCl_3$) δ 6.46 (s, 2H), 3.84 (s, 6H), 3.81 (s, 3H), 3.74 – 3.69 (m, 2H), 3.22 (dd, *J* = 8.2, 7.1 Hz, 2H), 2.91 (t, *J* = 7.7 Hz, 2H), 2.56 – 2.51 (m, 2H), 1.82 (ddd, *J* = 8.3, 4.1, 2.0 Hz, 4H).

^{13}C NMR (101 MHz, Acetone) δ 175.31, 172.91, 153.35, 137.21, 136.63, 105.79, 59.51, 55.42, 43.59, 41.10, 34.40, 31.34, 22.15, 20.02.

HRMS *m/z* (ESI+) $C_{17}H_{23}NO_5$ ([M+H]+) calculated 323.1576, found 323.1580

The spectroscopic data are in good agreement with literature values.[3]

(*E*)-2-cyano-3-(3,4,5-trimethoxyphenyl)acrylic acid (12)

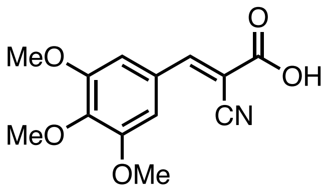

To a solution of *tert*- butyl 2-cyanoacetate (661 mg, 4.63 mmol, 1.0 equiv.) in dry DCM (15.7 mL) over activated molecular sieves (5 g) under argon atmosphere was added 3,4,5-trimethoxybenzaldehyde (1 g, 5.1 mmol, 1.1 equiv.) and piperidine (30 μL, 0.30 mmol, 0.07

equiv.). The mixture was stirred at room temperature until total consumption of *tert*-butyl 2-cyanoacetate was observed by TLC. The mixture was then quenched by the addition of aqueous solution saturated of NH_4Cl (120 mL) and then the aqueous phase was extracted with EtOAc (3 x 48 mL) and dried (Na_2SO_4). After removal of the solvent, purification by flash column chromatography (22% EtOAc/Petrol) gave tert-butyl (*E*)-2-cyano-3-(3,4,5-trimethoxyphenyl)acrylate as a bright yellow solid (752.8 mg, 2.36 mmol, 51%). This residue was dissolved in 23 mL of TFA and stirred at room temperature for 1.5 h. The TFA was evaporated under reduced pressure. The mixture was triturated with Et_2O, the residue was evaporated under reduced pressure (3 times). Filtration gave the corresponding acid **12** as a yellow oil (449.4 mg, 1.7 mmol, 37%).

^{1}H NMR (600 MHz, $CDCl_3$) δ 8.21 (s, 1H), 7.34 (s, 2H), 3.98 (s, 3H), 3.93 (s, 6H), 3.88 (s, 1H).

^{13}C NMR (126 MHz, Acetone) δ 163.8, 155.6, 154.5, 143.7, 127.8, 116.9, 109.7, 102.4, 60.9, 56.6.

(*E*)-2-(2-oxopiperidine-1-carbonyl)-3-(3,4,5-trimethoxyphenyl)acrylonitrile HKC54 (14)

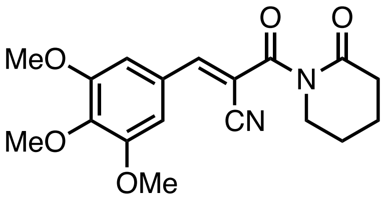

To a solution of (*E*)-2-cyano-3-(3,4,5-trimethoxyphenyl)acrylic acid (68.2 mg, 0.26 mmol, 1 equiv.), in freshly distilled THF (1.7 mL) was added triethylamine (30 μL, 0.21 mmol, 0.82 equiv.). Pivaloyl chloride (25.2 μL, 0.21 mmol, 0.79 equiv.) was added at –20 °C and the reaction mixture was stirred for 45 min. To a separate solution of piperidin-2-one (30.9 mg, 0.31 mmol, 1 equiv.) in freshly distilled THF (1.7 mL) was added *n*-BuLi (0.23 mL, 0.37 mmol, 1.2 equiv.) at –78 °C under argon and the reaction was stirred for 45 min. Then, anhydride prepared from the above step was added and the reaction mixture was stirred for 1 h. The reaction mixture was quenched with saturated NH_4Cl (0.6 mL), extracted with ethyl acetate (2 × 3 mL), the organic layer was separated and washed with sat. NaCl (2 × 2 mL) and dried over anhydrous Na_2SO_4. The residue was evaporated *in vacuo* to give a crude product which was purified by flash column chromatography on silica gel (3:2 Petrol/EtOAc) to yield **14** as a bright yellow oil (52 mg, 0.15 mmol, 58%).

R_f 0.19 (3:2 Petrol/EtOAc).

^{1}H NMR (600 MHz, $CDCl_3$) δ 7.82 (s, 1H), 7.24 (s, 2H), 3.94 (s, 3H), 3.91 (s, 6H), 3.74 (d, *J* = 6.1 Hz, 2H), 2.72 – 2.65 (m, 2H), 1.96 (d, *J* = 3.3 Hz, 4H).

^{13}C NMR (126 MHz, $CDCl_3$) δ 184.58, 174.02, 168.26, 153.23, 151.73, 142.21, 127.19, 116.37, 108.25, 107.50, 61.10, 56.28, 46.76, 34.41, 22.60, 21.46.

(*E*)-6-(3-(3,4,5-trimethoxyphenyl)acryloyl)cyclohex-2-en-1-one PL-0N (16)

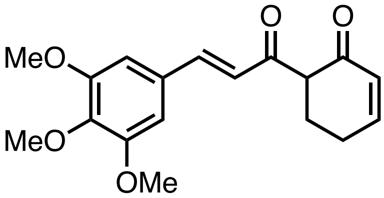

To a solution of cinnamic acid (256 mg, 1.07 mmol, 1 equiv.), in freshly distilled THF (6 mL) was added triethylamine (123 µL, 0.88 mmol, 0.82 equiv.). Pivaloyl chloride (105 µL, 0.85 mmol, 0.79 equiv.) was added at –20 °C and the reaction mixture was stirred for 45 min. To a separate solution of cyclohex-2-en-1- one (121 µL, 1.26 mmol, 1.2 equiv.) in freshly distilled THF (6 mL) was added LDA (692 µL of 2.0 M solution in THF, 1.38 mmol, 1.32 equiv.) dropwise at –78 °C under argon and the reaction was stirred for 45 min. Then, anhydride prepared from the above step was added and the reaction mixture was stirred for 1 h. The reaction mixture was quenched with saturated NH_4Cl (2.5 mL), extracted with ethyl acetate (2 × 10 mL), the organic layer was separated and washed with sat. NaCl (2 × 6 mL) and dried over anhydrous Na_2SO_4. The residue was evaporated *in vacuo* to give a crude product which was purified by flash column chromatography on silica gel (3:2 Petrol/EtOAc) to yield **16** as bright yellow crystals (210 mg, 0.66 mmol, 63%).

R_f 0.19 (3:2 Petrol/EtOAc).

^{1}H NMR (400 MHz, $CDCl_3$) δ 15.76 (d, *J* = 1.3 Hz, 1H), 7.54 (d, *J* = 15.5 Hz, 1H), 6.86 – 6.77 (m, 2H), 6.75 (s, 2H), 6.15 (dt, *J* = 9.9, 1.9 Hz, 1H), 3.90 (s, 6H), 3.87 (s, 3H), 2.75 (t, *J* = 7.2 Hz, 2H), 2.40 (tdd, *J* = 7.4, 4.3, 1.9 Hz, 2H).

^{13}C NMR (101 MHz, $CDCl_3$) δ 190.1, 169.8, 153.4, 146.9, 139.6, 139.0, 131.2, 129.8, 105.8, 105.0, 60.9, 56.2, 24.6, 22.0.

The spectroscopic data are in good agreement with literature values.[4]

2-(3-(but-3-yn-1-yl)-3H-diazirin-3-yl)ethyl 4-methylbenzenesulfonate (17)

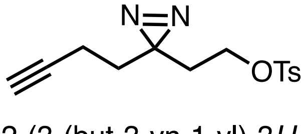

2-(3-(but-3-yn-1-yl)-3*H*-diazirin-3-yl)ethan-1-ol (185 µL, 1.45 mmol, 1 equiv.) was dissolved in CH_2Cl_2 (1 mL) at 0 °C. After the addition of triethylamine (605 µL, 4.34 mmol, 3 equiv.), and

DMAP (4 mg) to the solution, a solution of *p*-toluenesulfonyl chloride (551.9 mg, 2.90 mmol, 2 equiv.) in CH_2Cl_2 (2 mL) was added dropwise over 5 min, and the mixture was stirred at room temperature for 18 h. After addition of 1 M aqueous HCl, the mixture was extracted twice with Et_2O. The organic layer was concentrated *in vacuo*, and the residue was purified by flash column chromatography on silica gel (Petrol/EtOAc = 20:1) to afford **17** as a yellow oil (389.8 mg, 1.33 mmol, 92%).

^{1}H NMR (400 MHz, Acetone) δ 7.88 – 7.80 (m, 2H), 7.55 – 7.47 (m, 2H), 3.98 (t, *J* = 6.1 Hz, 2H), 2.47 (s, 3H), 2.38 (t, *J* = 2.7 Hz, 1H), 1.98 (td, *J* = 7.5, 2.6 Hz, 2H), 1.81 (t, *J* = 6.1 Hz, 2H), 1.59 (t, *J* = 7.5 Hz, 2H).

^{13}C NMR (101 MHz, Acetone) δ 205.2, 145.2, 133.1, 130.0, 127.9, 82.4, 69.8, 65.3, 32.2, 31.8, 26.0, 20.6, 12.5.

HRMS *m/z* (ESI+) $C_{14}H_{16}N_2O_3S$ ([M+H]+) calculated 292.0882, found 292.0873.

The spectroscopic data are in good agreement with literature values.[5]

(*E*)-1-(3-(4-hydroxy-3,5-dimethoxyphenyl)acryloyl)piperidin-2-one (18)

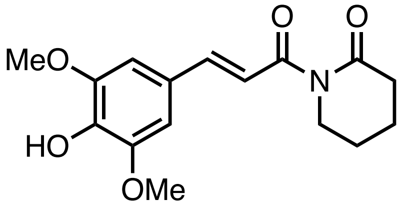

HKC20/**7** (116 mg, 0.36 mmol, 1 equiv.) was dissolved in CH_2Cl_2 (2.6 mL). Aluminium chloride (342 mg, 2.54 mmol, 7 equiv.) was added portion-wise at 0°C. The reaction was then warmed to room temperature and stirred for 1 h. The reaction was quenched with saturated aqueous NH_4Cl solution (2 mL), extracted with CH_2Cl_2 (2 x 8 mL), the organic layer was separated and washed with sat. NaCl (2 × 8 mL) and dried over anhydrous Na_2SO_4. The residue was evaporated *in vacuo* to give a crude product that was used in the next step without further purification to yield **18** as a pale yellow solid (55.8 mg, 0.18 mmol, 50%).

^{1}H NMR (500 MHz, Acetone) δ 7.63 – 7.51 (m, 1H), 7.32 (dd, *J* = 15.5, 7.8 Hz, 1H), 6.96 (s, 2H), 3.89 (s, 6H), 3.75 (ddd, *J* = 6.2, 3.9, 1.9 Hz, 2H), 2.60 – 2.54 (m, 2H), 1.89 (dq, *J* = 6.1, 3.3 Hz, 4H).

^{13}C NMR (126 MHz, Acetone) δ 173.2, 169.2, 148.1, 142.6, 120.0, 105.9, 55.8, 44.2, 34.5, 22.4, 20.5.

1-(3-(4-hydroxy-3,5-dimethoxyphenyl)propanoyl)piperidin-2-one (19)

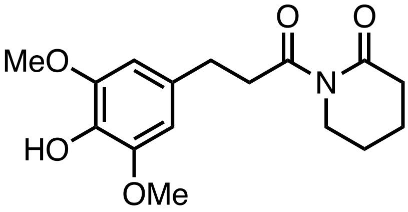

18 (25 mg, 0.082 mmol, 1 equiv.) was dissolved in 1:3 toluene/CH_2Cl_2 (0.9 mL). Pd/C catalyst (2.5 mol %) followed by acetic acid (19 µL, 0.33 mmol, 4 equiv.) were added in single portions. $NaBH_4$ (25 mg, 0.66 mmol, 8 equiv.) was added in a single portion directly to the stirring heterogeneous solution (Note: addition of the $NaBH_4$ causes the rapid evolution of small hydrogen gas bubbles. Avoid open flames). The mixture was left to stir in the open air at room temperature for 1 h. The reaction was quenched with 0.1 M HCl until no further hydrogen evolution was observed. The solution was then made basic using $NaHCO_3$ and organic layers extracted with CH_2Cl_2 (2 x 2 mL), dried over anhydrous $MgSO_4$ and filtered through celite. The residue was evaporated *in vacuo* to give a crude product that was used in the next step without further purification to yield **19** as a pale yellow solid (7 mg, 0.02 mmol, 30%).

^{1}H NMR (400 MHz, Acetone) δ 6.53 (s, 2H), 3.88 (s, 1H), 3.80 (s, 8H), 3.67 (t, *J* = 1.5 Hz, 1H), 3.12 (dd, *J* = 8.5, 7.0 Hz, 2H), 2.84 – 2.79 (m, 5H), 2.51 – 2.45 (m, 2H), 1.85 – 1.76 (m, 4H).

^{13}C NMR (101 MHz, Acetone) δ 175.4, 172.9, 147.7, 131.9, 105.9, 55.7, 43.6, 41.4, 34.4, 31.1, 22.2, 20.0.

HRMS *m/z* (ESI+) $C_{16}H_{21}NO_5$ ([M+H]+) calculated 307.1420, found 307.1409.

1-(3-(4-(2-(3-(but-3-yn-1-yl)-3H-diazirin-3-yl)ethoxy)-3,5-dimethoxyphenyl)propanoyl)piperidin-2-one Photo-HKC22 (20)

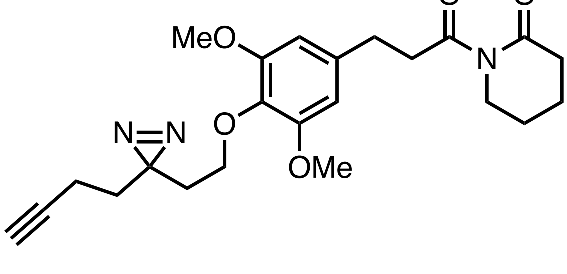

A solution of **19** (7 mg, 0.023 mmol, 1 equiv.) in anhydrous DMF (0.2 mL) was cooled to 0°C. Potassium *tert*-butoxide (5.11 mg, 0.046 mmol, 2 equiv.) was added and the mixture was stirred for 5 min. 2-(3-(but-3-yn-1-yl)-3H-diazirin-3-yl)ethyl 4-methylbenzenesulfonate/**17** (13.3 mg, 0.046 mmol, 2 equiv.) was subsequently added and the reaction mixture was stirred for 18 h at room temperature. The reaction was quenched with H_2O (2 mL), extracted with Et_2O

(2 x 10 mL) and the combined organic layers were separated, washed with sat. NaCl (2 × 6 mL) and dried over anhydrous Na_2SO_4. The residue was evaporated *in vacuo* to give a crude product which was purified by flash column chromatography on silica gel (3:2 Petrol/EtOAc) to yield **20 (Photo-HKC22)** as a yellow oil (2.2 mg, 0.0051 mmol, 23% yield).

^{1}H NMR (400 MHz, Acetone) δ 6.95 (s, 2H), 3.83 (d, *J* = 6.3 Hz, 2H), 3.81 (s, 6H), 3.72 – 3.62 (m, 2H), 3.14 (dd, *J* = 8.5, 7.0 Hz, 2H), 2.84 (dd, *J* = 8.4, 7.1 Hz, 2H), 2.53 – 2.44 (m, 2H), 2.36 (t, *J* = 2.7 Hz, 1H), 2.12 – 2.07 (m, 2H), 1.83 – 1.77 (m, 6H), 1.71 (t, *J* = 6.3 Hz, 2H).

HRMS *m/z* (ESI+) $C_{23}H_{29}N_3O_5$ ([M+H]+) calculated 427.2107, found 427.2123.

(*E*)-1-(3-(4-hydroxy-3,5-dimethoxyphenyl)acryloyl)-5,6-dihydropyridin-2(1H)-one (21)

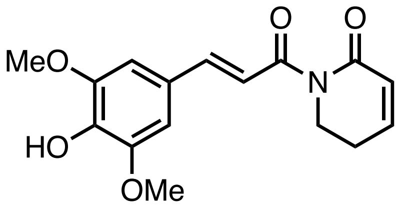

PL (81 mg, 0.26 mmol, 1 equiv.) was dissolved in CH_2Cl_2 (1.85 mL). Aluminium chloride (241 mg, 1.8 mmol, 7 equiv.) was added portion-wise at 0°C. The reaction was then warmed to room temperature and stirred for 1 h. The reaction was quenched with saturated aqueous NH_4Cl solution (1 mL), extracted with CH_2Cl_2 (2 x 4 mL), the organic layer was separated and washed with sat. NaCl (2 × 6 mL) and dried over anhydrous Na_2SO_4. The residue was evaporated *in vacuo* to give a crude product which was purified by column chromatography on silica gel (1:4 Petrol/EtOAc) to yield **21** as a pale yellow crystalline solid (61 mg, 0.2 mmol, 78%).

^{1}H NMR (400 MHz, Acetone) δ 7.59 (d, *J* = 15.5 Hz, 1H), 7.35 (d, *J* = 15.5 Hz, 1H), 7.06 (dt, *J* = 9.7, 4.2 Hz, 1H), 6.97 (s, 2H), 5.96 (dt, *J* = 9.7, 1.9 Hz, 1H), 3.96 (t, *J* = 6.4 Hz, 2H), 3.88 (s, 6H), 2.51 (tdd, *J* = 6.3, 4.2, 1.9 Hz, 2H).

^{13}C NMR (101 MHz, Acetone) δ 169.3, 166.3, 149.0, 147.1, 144.0, 139.4, 126.9, 126.0, 120.6, 106.8, 56.7, 42.4, 25.4.

HRMS *m/z* (ESI+) $C_{16}H_{17}NO_5$ ([M+H]+) calculated 303.1107, found 303.1108.

(*E*)-1-(3-(4-(2-(3-(but-3-yn-1-yl)-3H-diazirin-3-yl)ethoxy)-3,5-dimethoxyphenyl)acryloyl)-5,6-dihydropyridin-2(1H)-one Photo-PL (22)

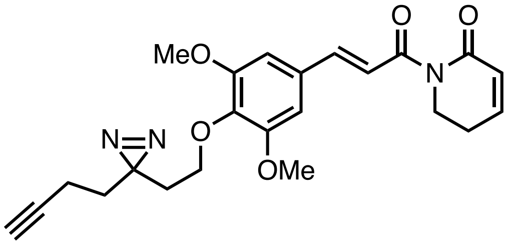

A solution of **21** (20 mg, 0.066 mmol, 1 equiv.) in anhydrous DMF (0.15 mL) was cooled to 0°C. Potassium *tert*-butoxide (14.8 mg, 0.13 mmol, 2 equiv.) was added and the mixture was stirred for 5 min. 2-(3-(but-3-yn-1-yl)-3H-diazirin-3-yl)ethyl 4-methylbenzenesulfonate/**24** (38.6 mg, 0.13 mmol, 2 equiv.) was subsequently added and the reaction mixture was stirred for 18 h at room temperature. The reaction was quenched with H_2O (2 mL), extracted with Et_2O (2 x 10 mL) and the combined organic layers were separated, washed with sat. NaCl (2 × 6 mL) and dried over anhydrous Na_2SO_4. The residue was evaporated *in vacuo* to give a crude product which was purified by column chromatography on silica gel (3:2 Petrol/EtOAc) to yield **22** as a yellow oil (2.7 mg, 0.0064 mmol, 10% yield).

^{1}H NMR (500 MHz, Acetone) δ 7.67 – 7.56 (m, 1H), 7.42 (d, *J* = 15.6 Hz, 1H), 7.10 (dt, *J* = 9.6, 4.1 Hz, 1H), 7.00 (s, 2H), 5.99 (dt, *J* = 9.7, 1.9 Hz, 1H), 3.99 (t, *J* = 6.4 Hz, 2H), 3.95 (d, *J* = 6.2 Hz, 2H), 3.93 (s, 6H), 2.53 (qdd, *J* = 8.7, 5.8, 2.3 Hz, 2H), 2.40 (t, *J* = 2.7 Hz, 1H), 2.16 – 2.09 (m, 2H), 1.83 (dd, *J* = 8.1, 7.2 Hz, 2H), 1.77 (t, *J* = 6.2 Hz, 2H).

^{13}C NMR (176 MHz, Acetone) δ 168.3, 165.4, 153.8, 146.4, 142.2, 138.9, 131.0, 125.0, 121.9, 105.6, 82.9, 69.5, 67.7, 55.7, 41.5, 33.9, 32.2, 26.9, 24.5, 22.4, 13.5, 12.8.

HRMS *m/z* (ESI+) $C_{23}H_{25}N_3O_5$ ([M+H]+) calculated 423.1794, found 423.1823.

2.4 Cell lines and cell culture

In this study, the following mammalian cell lines were used: human glioblastoma (U-87 MG, U-251, U-251 TRPV2 KD, DBTRG-05MG and YKG-1) cell lines; human acute myeloid leukaemia (MOLM-13 and HEL) cell lines; human cervix carcinoma (HeLa) cells; human oestrogen/progesterone/HER2 receptor-negative (triple-negative) breast carcinoma (MDA-MB-231) cells; human breast adenocarcinoma (MCF-7) cells and human embryonic kidney (HEK293T) cells. U-87 MG (ATCC-HTB-14™) cells were obtained from ATCC. All other cell lines were obtained from the Instituto de Medicina Molecular in Lisbon. All cells were cultured in a standard humidified incubator at 37 °C, 5% CO_2 and were tested for mycoplasma contamination on a regular basis. HEK293T, U-87 MG, U-251, U-251 TRPV2 KD, YKG-1, HeLa, MCF-7 and DBTRG-05 MG cells were subcultured in DMEM GlutaMAX™ (Gibco™)

supplemented with 10% fetal bovine serum (FBS, heat inactivated; Gibco™). MDA-MB-231 cells were subcultured in McCoy's 5A (Gibco™) supplemented with 10% FBS (heat inactivated, Gibco™). MOLM-13 and HEL cells were subcultured in RPMI supplemented with 10% FBS (heat inactivated, Gibco™). All cell lines were cultured without antibiotics.

2.5 GSH/GSSG-Glo™ assay

MCF-7 cells were seeded in 96-well plate at a density of 7000 cells/well and allowed to attach overnight. Compounds PL, HKC20, HKC22, HKC54 and PL-0N were synthesised and characterised as described (**section 2.3**). Menadione (>98%; Sigma Aldrich) was used as a positive control. Cells were treated for 6 h with 20 µM of each compound (PL, HKC20, HKC22, HKC54, PL-0N and menadione) or DMSO vehicle control. The media was removed and wells washed with DPBS. The GSH/GSSG-Glo™ kit (Promega) was used to measure total glutathione (GSH) according to the manufacturer's instructions. The luminescence was read on a BMG LABTECH CLARIOstar microplate reader. Data was analysed using GraphPad Prism version 9.5.0. Data are expressed as mean ± SD (n = 6). Statistical significance was calculated with one-way ANOVA and Tukey's multiple comparisons test. ****P < 0.0001, ***P < 0.001.

2.6 Fluorescence imaging of oxidative stress

MCF-7 cells were seeded at a density of 30,000 cells/well in µ-ibidi 8-well glass bottom slides and allowed to attach overnight. Compounds PL, HKC20, HKC22 and HKC54 were synthesised and characterised as described (**section 2.3**). Menadione (>98%; Sigma Aldrich) was used as a positive control. Cells were treated for 4 h with 20 µM of each compound (PL, HKC20, HKC22, HKC54 and menadione) or DMSO vehicle control. The CellROX® Deep Red (C10422; Thermo Fisher Scientific) reagent was added to all wells at a final concentration of 5 µM and the slides were incubated for 30 min at 37 °C. The cells were subsequently washed 3 times with DPBS and cell fixing solution was added (3.7% paraformaldehyde in PBS). The cells were fixed for 15 min at rt after which the wells were washed twice with DPBS. The nuclei were stained with 1X Hoechst 33342 (1000X in DPBS) and were left in this solution for subsequent fluorescence imaging with an EVOS M5000 microscope (Cy5 filter). Images were analysed using ImageJ.

2.7 CellTiter-Glo® luminescent cell viability assay

Cells were seeded at 20,000 per well (10,000 per well for HeLa cells) in 96-well white flat bottom TC-treated microplates (Falcon®) and allowed to attach overnight. The perimeter wells were not used and were filled with media/PBS to avoid evaporation gradient across the plate. Compounds PL, HKC20, HKC22 and HKC54 were synthesised and characterised as described (**section 2.3**). After addition of compounds or DMSO vehicle control, plates were incubated for 48 h. DMSO was maintained at an in-plate concentration of 0.1%. CellTiter-Glo® Luminescent Cell Viability Assay (Promega) was used according to the manufacturer's instructions. Luminescence was measured using a SpectraMax i3x plate reader, and signal intensity was calculated relative to in-plate DMSO control wells. Data was analysed using GraphPad Prism version 9.5.0. Data are expressed as mean ± SD (n = 3).

2.8 Fluorescence imaging of intracellular calcium in HEK293T cells

HEK293T cells were cultured to 30% confluency in DMEM containing 10% FBS (GIBCO, Grand Island, NY), 1% GlutaMAX (GIBCO, Grand Island, NY), and 1% pen-strep (GIBCO, Grand Island, NY) in 10% CO_2 at 37 °C. For transfection, cells were seeded in µ-ibidi 8 well plates and overnight and transfected with the plasmids hTRPV2-flag-RFP pcDNA3.1(+) and TRPV2 mutant variants, using Fugene HD Reagent (Promega) as previously described.[6] As control cells were also transfected with RFP pcDNA3.1(+) empty vector. After 48 h of incubation, cells were subjected to calcium imaging. Intracellular calcium measurements were performed with Fura-2 AM (Life Technologies) and modified from previously described studies.[7] In short, 1 h before the measurement, cells were loaded with 5 µM Fura-2 AM for 45 min. Cell medium was replaced by Tyrod's solution (119 mM NaCl, 5 mM KCl, 2 mM $CaCl_2$, 2 mM $MgCl_2$, 6 g/L glucose, 25 mM HEPES pH 7.4). Simultaneously, the respective treatment was added to the cells (final concentration of 1% DMSO (control), 5 µM PL and derivatives HKC20, HKC22 and HKC54, and 10 µM tranilast) and cells were allowed to re-adjustment for 15 min. Fura-2 AM emissions from 340 nm and 380 nm excitation were recorded for 1 min before TRPV2 was activated by addition of 4 µM cannabidiol (CBD). Samples were imaged using an inverted microscope with epifluorescent optics (Axiovert 135TV, Zeiss) and equipped with a high-speed multiple excitation fluorimetric system (Lambda DG4, with a 175W Xenon arc lamp). Data were recorded by a CDD camera. Images were collected and analysed using MetaFluor Fluorescence Ratio Imaging Software (Molecular Devices). The statistical analysis was performed with astatsa.com. Experiments were carried out at room temperature. For further analysis, the 30 to 40 most responsive cells per image were selected manually, based on the increases in $[Ca^{2+}]_i$ (from highest to lowest). Experiments were performed at least in

triplicate. After calcium imaging acquisition, the cells were immediately fixed with 4% paraformaldehyde for 15 min. Cells were washed thrice with PBS and embedded in Fluoromount G. Samples were imaged using the LSM Zeiss 880 microscope.

2.9 Primary cultures of rat DRG nociceptors

Neonatal dorsal root ganglia (DRGs) from Wistar rats (3–5 days-old) were isolated and digested with 0.25% (w/v) collagenase (type IA) in DMEM GlutaMax with 1% (v/v) penicillin/streptomycin (P/S) solution for 1 h (37 °C, 5% CO_2, ThermoScientific incubator) as previously described.[8] After digestion, DRGs were mechanically dissociated. Single cell suspension was passed through a 100 µm cell strainer and washed with DMEM GlutaMax with 10% (v/v) fetal bovine serum (FBS) and 1% (v/v) P/S. Cells were seeded on 12 mm cover-glass slides. After 1 h, medium was replaced with DMEM GlutaMax, 10% (v/v) FBS and 1% (v/v) P/S, supplemented with mouse 2.5S NGF 50 ng/mL and 1.25 µg/mL cytosine arabinoside (37 °C, 5% CO_2). All experiments were performed after 48 h cell seeding.[9] All cell culture procedures were performed in a laminar flow cabinet (Model Telstar AV-100).

2.10 Fluorescence imaging of intracellular calcium in DRG nociceptors

Calcium experiments were conducted using the non-ratiometric fluorescent probe fluo4-AM (F14201, Thermo Fisher Scientific). Cells were incubated for 60 min at 37°C with a loading buffer comprising 6 mg/mL fluo4-AM and 0.2% w/v pluronic acid (F-127, Thermo Fisher Scientific) dissolved in HBSS (HANKS balanced salt solution) (NaCl 140 mM, KCl 3 mM, CaCl2 2.4 mM, MgCl2 1.3 mM, HEPES 10 mM, and glucose mM, adjusted to pH 7.4 with NaOH 1M). To remove excess fluorophore, cells were washed with HBSS for at least 20 min. Calcium imaging experiments were conducted using an inverted microscope (Axiovert 200/B, ZEISS) equipped with a Hamamatsu Flash 4.0 LT camera (C11440-42U30, Hamamatsu, Sunayama-cho, Japan). Cells were identified using bright field microscopy, and regions of interest were manually delineated. Fluo4 was excited at 480 nm (with 200-400 ms of excitation time) using a rapid-gating shutter (lambda-shutter 10/2 Sutter instruments, Novato, USA), and images were captured every 3 seconds. Mean fluorescence intensity for each cell at each time point was measured using HCimage DIA software (Hamamatsu Photonics). Experiments were conducted at 22-26°C.

The size of calcium transients was determined by measuring the maximum values after agonist application and subtracting the fluorescence values at the resting state. All the antagonists were applied for a time of 1min before the agonist application and the response was normalized on the desensitization obtained in the control conditions (agonist application).

To ensure comparability, these results were normalized by the positive control in each experiment. Calcium transients were considered positive responses if fluorescence increases exceeded 0.2 arbitrary units. Substances dissolved in HBSS were applied through a perfusion system controlled with automatic valve clamps (PC-16 Bioscience Tools, S. Diego, USA) for periods of 15 to 30 seconds. Cells were washed with extracellular solution between calcium responses for at least 300 seconds to ensure recovery of basal fluorescence levels. At the end a perfusion of 15s of KCl was applied to ensure the viability of sensory neurons.

2.11 Electrophysiological recordings with DRG nociceptors

Patch-clamp recordings were performed on dorsal root ganglion (DRG) nociceptors. Two days post-seeding on 12 mm Ø glass coverslips coated with poly-L-lysine solution and Laminin (Sigma Aldrich), whole-cell patch-clamp recordings were conducted on sensory DRG neurons harvested from adult mice. For intracellular recording, the pipette solution comprised (in mM): 4 NaCl, 110 K gluconate, 1 $CaCl_2$, 30 KCl, 2 $MgCl_2$, 10 HEPES, 4 ATP, 0.4 GTP, and 10 EGTA, adjusted to a pH of 7.2 with KOH. Extracellular solution, for bath application, consisted of (in mM): 140 NaCl, 4 KCl, 2 $CaCl_2$, 2 $MgCl_2$, 10 HEPES, 5 glucose, and 20 mannitol, with a pH of 7.4 adjusted using NaOH. To account for channel desensitization, two consecutive agonist pulses (P1 and P2) were administered. The protocol consists of the application of two brief 30-second pulses considered as P1 and P2, respectively, of the agonist (400 μM probenecid) via a continuous perfusion system. Following each stimulus application, cells underwent a thorough wash with external solution for 3 min to ensure proper recovery for subsequent trials. Prior to P2, the treated cells were perfused with HKC54, HKC22, or HKC20 (5 μM), for a duration of 1 min. Throughout the procedure, all measurements were conducted at approximately 22°C. The TRPV2 responses were assessed by calculating the P2/P1 ratio, representing the current evoked by second agonist pulse normalized to the first agonist pulse. This evaluation was performed both in the absence (control, n = 6) and presence of HKC22, HKC20, and HKC54 (n = 6) prior to the second pulse of TRPV2 agonist. Data were expressed as mean ± SEM. **TRPV1**: P2/P1 ratio denoting TRPV1 current evoked by each pulse of agonist capsaicin (0.1 μM), normalised to first vanilloid pulse, in the absence (control, n = 4) and the presence of 10 μM PL, HKC22, HKC20 and HKC54 (n= 5) before the second pulse of capsaicin. Two consecutive agonist pulses (P1 and P2) are applied to account for channel desensitisation. **TRPA1:** P2/P1 ratio denoting TRPA1 current evoked by each pulse of agonist AITC (100 μM), normalised to first vanilloid pulse, in the absence (control, n = 4) and the presence of 10 μM PL, HKC22, HKC20 and HKC54 (n= 5) before the second pulse of AITC. Two consecutive agonist pulses (P1 and P2) are applied to account for channel

desensitisation. **TRPM8**: P2/P1 ratio denoting TRPM8 current evoked by each pulse of agonist menthol (100 µM), normalised to first vanilloid pulse, in the absence (control, n = 4) and the presence of 10 µM PL, HKC22, HKC20 and HKC54 (n= 5) before the second pulse of menthol. Two consecutive agonist pulses (P1 and P2) are applied to account for channel desensitisation. All data are expressed as mean ± SEM. Data was analysed using an unpaired, two-tail Student's t-test. ****$p < 0.0001$.

2.12 Photoaffinity labelling methods

2.12.1 Photoaffinity labelling probe concentration determination for in-gel fluorescence

HEK293T cells were seeded at a density of 0.6M/mL in two 6-well plates and allowed to attach overnight. The media was replaced with serum-free media when various concentrations of Photo-HKC22 (5, 10, 20, 25 and 40 µM) and DMSO vehicle were added (0.1% DMSO in each well). Cells were treated for 1 h. Subsequently, one plate without its lid was irradiated on ice for 5 min with a 365 nm LED lamp (HCK1012-01-006; EvoluChem™), 5 cm from light source. The other plate was not irradiated to serve as a labelling control. Cells were washed with ice-cold PBS and lysed in 200 µL of lysis buffer (0.5% triton-x-100 in PBS supplemented with 1% EDTA-free protease and phosphatase inhibitor cocktail (PPC1010; Sigma-Aldrich) and 1% DNAse II). Protein quantification was performed using the Pierce™ BCA assay (23225; Thermo Fisher Scientific) and all lysates were diluted/normalised to 1 mg/mL with PBS. A click master mix was prepared (50 mM $CuSO_4$, 1.25 mM rhodamine-azide, 50 mM TCEP.HCl and 1.7 mM TBTA). Per sample, 6 µL of master mix was added to 50 µL of lysate. The click reaction proceeded for 1 h at rt and was quenched with 20 µL of 4X LDS loading buffer and 2 µL of β-mercaptoethanol. Proteins were separated by SDS PAGE electrophoresis using NuPAGE 4-12% Bis-Tris gels (Invitrogen) for 1 h at 180V in 1 x MES SDS running buffer (Invitrogen). Gels were imaged using a ChemiDoc MP and Coomassie staining was performed using InstantBlue® (ab119211; Abcam).

2.12.2 Photoaffinity labelling competition study for in-gel fluorescence

To compete probe Photo-HKC22 with parent compound HKC22, the same procedure for photoaffinity labelling was performed (**section 2.12.1**) but the cells were first pre-treated with various excess concentrations of HKC22 (400, 200, 100, 50, 25, 12.5, 6.25, 3.13, 1.56, 0.78, 0.39 µM) or DMSO for 45 min.[10] Afterwards, the cells were treated with a fixed concentration

of Photo-HKC22 (20 μM) for 1 h and the lysis, click reaction and gel electrophoresis procedures followed.

2.12.3 In-situ labelling of cells with photoaffinity probe for proteomics

Competitor compounds were dissolved in DMSO to 200 mM and diluted in 3 mL serum-free DMEM media to a final treatment concentration of 500 μM unless otherwise stated. HEK293T cells (90-100% confluent in 10 cm plates) were washed with pre-warmed PBS. Next, the 3 mL of diluted competitor solution was added to the cells, and they were incubated for 30 min in the cell incubator. 3 μL 25 mM stock solution of the FFF probe (in DMSO) was prepared in 15 mL tubes. The competitor solution was taken off the cells, mixed with the FFF probe and returned to the cells. Cells were incubated 5 min and afterwards cells were illuminated with UV light (UVP Crosslinker, AnalytikJena, 365 nm wavelength, 100% intensity) for 5 min at 4°C. Cell pellets were harvested and washed twice with 1 mL ice-cold PBS, snap frozen with liquid nitrogen in 1.5 mL LoBind tubes and stored at -80°C until further processing.

2.12.4 Preparation of probe-labeled proteome for MS-based protein analysis

16 samples were processed in parallel to fill all channels of a TMTpro 16plex. Frozen cell pellets were resuspended in 200 μL lysis buffer (1x PBS, 1% SDS, 2 mM $MgCl_2$, 1x Halt Protease inhibitor cocktail, 50 Units Benzonase), vortexed and incubated at 37°C and 300 rpm shaking for 30 min. The samples were centrifuged for 30 min at 18,000 x g and +4°C and supernatants were transferred into fresh 1.5 mL lo-bind tubes on ice. Total protein concentration was determined with the Pierce 660 nm Protein Assay with Ionic Detergent Compatibility Reagent (IDCR). 750 μg proteome were diluted in 150 μL lysis buffer (5 μg/μL) in 5 mL LoBind tubes and a biotin handle was attached via Cu(I)-catalyzed azide-alkyne cycloaddition (CuAAC). To do so, following reagents were added to each sample: 450 μl phosphate buffer (61.34 mM K_2HPO_4, 38.21 mM KH_2PO_4, pH 7.2), 25 μL biotin-PEG3-azide (5 mM in DMSO, final concentration 170 μM), 25 μL of a 1:2 premix of $CuSO_4$ (20 mM in H_2O, final concentration 230 μM) and THPTA (50 mM in H_2O, final concentration 1.15 mM), 35 μL aminoguanidine HCl (100 mM in H_2O, final concentration 5 mM), 35 μL sodium ascorbate (100 mM in H_2O, final concentration 5 mM). Samples were vortexed and incubated on a rotator at 25°C for 1 h. 3 ml acetone (cooled at -80°C for 30 min in advance) was added, samples were vortexed and stored at -80°C for 30 min followed by centrifugation at 4°C and 18,000 x g for 30 min. The supernatant was decanted carefully, tubes were dabbed on paper and left drying for 30 min. Protein pellets were stored at -80°C until further processing.

Protein pellets were resuspended in 300 µL 1% SDS by pipetting, sonication at +4°C (Bioruptor pico, Diagenode) and heating to 56°C. Reduction was done by addition of 30 µL TCEP (50 mM stock solution in H_2O, final concentration 4.5 mM), vortexing and incubation on a rotator at 56°C for 1 h. The pH was adjusted with 80 µL 1 M HEPES pH 7.5 and samples were alkylated by addition of 45 µL freshly prepared iodoacetamide (200 mM stock solution in H_2O, final concentration 20 mM), vortexing and incubation on rotator at 25°C for 30 min. Next, the whole sample was added to 100 µL slurry of streptavidin agarose resin (washed twice before with 4 ml PBS) and 1.35 mL PBS in a 2 ml LoBind tube. Samples were incubated on rotator at 25°C for 1 h, spun down and the complete supernatant was taken off and discarded. Minispin columns (Bio-Rad) were equilibrated on vacuum manifold with 2x 0.5 ml Wash buffer 1 (0.2% SDS in 1x PBS) and the beads were transferred with 2x 0.5 ml Wash buffer 1 into these columns. Using a multidispense pipette the beads were washed 16 times with 0.5 ml Wash buffer 2 (8 M urea in 1x PBS) and 4 times with 0.5 ml 1x PBS. The beads were transferred with 2x 0.5 ml Digestion buffer (50 mM ammonium bicarbonate, 200 mM Guanidine hydrochloride, 1 mM Calcium chloride, in HPLC grade water) into fresh 1.5 ml LoBind tubes, spun down, the supernatant was discarded. 250 µL fresh Digestion buffer and 10 µL Trypsin (0.1 µg/µl, total 1 µg per sample) were added and samples were incubated at 37°C and rotation overnight (~14 h).

The beads were spun down and the supernatant containing the peptides was transferred into fresh 1.5 mL LoBind tubes. Beads were resuspended in 200 µL HPLC grade water, spun down again and the supernatant was combined with the initial supernatant. Peptides clean-up was done with self-made stage tips. The stage tip columns were assembled by punching out 1 mm C18 material from Empore C18 disks using a blunt syringe needle and pushed into the tip of a 200 µL pipette tip. On top, 24 µl oligo R3 solution (15 mg/mL in acetonitrile) were applied, followed by 1 min centrifugation at 1,000 x g inside of 2 mL collection tube. The C18 material was activated by washing twice with 100 µL acetonitrile (1 min centrifugation at 1,000 x g) and the column was equilibrated twice with 200 µL 0.1% TFA (3 min centrifugation at 1,000 x g). The peptide samples were acidified with 16 µL 30% TFA (~1% final), vortexed and spun down. Samples were loaded in fractions (max. capacity 250 µL at once, centrifuged at 1,000 x g for 3 min each). Columns were washed with 200 µL 0.1% TFA (3 min centrifugation at 1,000 x g). Peptides were eluted into fresh 1.5 mL LoBind tubes with 2x 50 µL Elution buffer (90% acetonitrile, 0.01% TFA, in HPLC grade water). The eluates were dried in vacuum centrifuge at 45°C until completely dry and stored at -20°C.

The peptide pellets were reconstituted in 15 µL 100 mM HEPES pH 8.5 (pharmaceutical standard in H_2O for HPLC). 4 µL of the respective TMTpro 16plex label (0.01 mg/µL in

acetonitrile) was added to each sample. Samples were vortexed, spun down and incubated at 25°C and 300 rpm for 1 h. The reaction was stopped by addition of 1.5 µL of 5% hydroxylamine solution (in H_2O for HPLC, prepared freshly from a 50% hydroxylamine stock solution). Samples were vortexed, spun down and incubated at 25°C and 300 rpm for 15 min. Pooling of full volumes of samples of respective TMTpro 16plex channels into a fresh 1.5 mL LoBind tube and on-tip high pH fractionation for 2D analysis was performed next. To do so, 1 mL of freshly prepared 20 mM ammonium formate (in HPLC grade water, pH 10 adjusted with 25% ammonia solution) was added to 320 µL of pooled sample. A self-made C18 column for fractionation was prepared: 1 mm C18 material from an Empore C18 disk was punched out using a blunt syringe needle and pushed into the tip of a 200 µL pipette tip. On top, 24 µL oligo R3 solution (15 mg/mL in acetonitrile) were applied, followed by 1 min centrifugation at 1,000 x g inside of 2 mL collection tube. The C18 material was activated by washing twice with 100 µL acetonitrile (1 min centrifugation at 1,000 x g) and the column was equilibrated twice with 200 µL of 20 mM ammonium formate pH 10 (3 min centrifugation at 1,000 x g). Samples were loaded in fractions (max. capacity 250 µL at once, centrifuged at 1,000 x g for 3 min each) and the columns were washed with 200 µL of 20 mM ammonium formate pH 10 (3 min centrifugation at 1,000 x g). Peptides were eluted in five fractions with fractionation buffers with acetonitrile and 20 mM ammonium formate pH 10 mixed at different ratios (16%, 20%, 24%, 28%, 80% acetonitrile, respectively). Each fraction was collected with 50 µL of the respective fractionation buffer (2 min centrifugation at 1,000 x g) followed by 20 µL of the same fractionation buffer (2 min centrifugation at 1,000 x g) in the same 1.5 mL LoBind tube. All five eluates were dried in a vacuum centrifuge at 45°C until complete dryness. Dried peptide pellets were stored at -20°C until they were reconstituted in 20 µL of 0.1% TFA for analysis by 2D-RP/RP Liquid Chromatography – Tandem Mass Spectrometry analysis (see below).

2.12.5 2D-RP/RP Liquid Chromatography – Tandem Mass Spectrometry analysis

Mass spectrometry analysis was performed on an Orbitrap Fusion Lumos Tribrid mass spectrometer coupled to a Dionex Ultimate 3000 RSLC nano system via a Nanospray Flex Ion Source interface. 10 µL peptide solution were loaded onto a trap column (PepMap 100 C18, 5 µm, 5 × 0.3 mm) at a flow rate of 10 µL/min using 0.1% TFA as loading buffer. After loading, the trap column was switched in-line with an Acclaim PepMap nanoHPLC C18 analytical column (2.0 *µ*m particle size, 75 *µ*m ID x 500 mm, catalog number 164942, Thermo Fisher Scientific, San Jose, CA). The column temperature was maintained at 50 °C. Mobile phase A consisted of 0.4% formic acid in water, and mobile phase B consisted of 0.4% formic acid in a mixture of 90% acetonitrile and 10% water. Separation was achieved using a four-

step gradient over 150 min at a flow rate of 230 nL/min (increase of initial gradient from 6% to 9% solvent B within 1 min, 9% to 30% solvent B within 146 min, 30% to 65% solvent B within 8 min, 65% to 100% solvent B within of 1 min and 100% solvent B for 6 minutes before equilibrating to 6% solvent B for 24 min before the next injection). In the liquid junction setup, electrospray ionization was enabled by applying a voltage of 1.8 kV directly to the liquid being sprayed, and non-coated silica emitter was used.

The mass spectrometer was operated in a data-dependent acquisition mode (DDA) and used a synchronous precursor selection (SPS) approach. For both MS2 and MS3 levels, a 400–1600 m/z survey scan in the Orbitrap at 120 000 resolution (FTMS1) was collected, the AGC target was set to 'standard' and a maximum injection time (IT) of 50 ms was applied. Precursor ions were filtered by charge state (2-5), dynamic exclusion (60 s with a ±10 ppm window), and monoisotopic precursor selection. Precursor ions for data-dependent MSn (ddMSn) analysis were selected using 10 dependent scans (TopN approach). A charge-state filter was used to select precursors for data-dependent scanning. In ddMS2 analysis, spectra were obtained using one charge state per branch (from z=2 to z=5) in a dual-pressure linear ion trap (ITMS2). The quadrupole isolation window was set to 0.7 Da and the collision-induced dissociation (CID) fragmentation technique was used at a normalized collision energy of 35%. The normalized AGC target was set to 200% with a maximum IT of 35 ms. During the ddMS3 analyses, precursors were isolated using SPS waveform and different MS1 isolation windows (1.3 m/z for z=2, 1.2 m/z for z=3, 0.8 m/z for z= 4 and 0.7 m/z for z = 5). Target MS2 fragment ions were further fragmented by high-energy collision induced dissociation (HCD) followed by orbitrap analysis (FTMS3). The normalized HCD collision energy was set to 45% and the normalized AGC target was set to 300% with a maximum IT of 100 ms. The resolution was set to 50 000 with a defined scanning range of 100 to 500 m/z. Xcalibur Version 4.3.73.11 and Tune 3.4.3072.18 were used to operate the instrument.

2.12.6 Peptide and protein identification and quantification

LC/MS data processing was performed with Thermo ProteomeDiscoverer 2.4, where all five .raw files of a given experiment were processed as fractions. Initially, a signal/noise threshold of 1.5 for all peaks was applied. After that, fragment spectra were searched via Sequest HT against the canonical human proteome (UniProt release 2022.11.12), a database of common contaminants (Proteome Discoverer Contaminants 2014) and the sequence of Streptavidin (B8YQ01). For generating database spectra, tryptic cleavage with max. 2 missed cleavages was applied. MS1 precursors were matched with 10 ppm tolerance, and a tolerance of 0.6 Da was applied for lower resolution ion-trap MS2 ions. The minimum peptide length was set to 6

residues. Oxidation (+15.995 Da) of Methionine was set as a dynamic amino acid modification. Dynamic N-Terminal modifications were set for Acetylation (+42.011 Da), Methionine-loss (-131.040 Da) and Methionine-loss plus Acetylation (-89.030 Da). Static Modifications were set for TMTpro labelling (+304.207 Da on N-Termini, +304.207 Da on Lysines) and Cysteine Alkylation (Carbamidomethylation, +57.021 Da). To allow for FDR-based Peptide Spectrum Match Validation by Percolator, spectra were searched against a concatenated database of real peptides and reverse-shuffled decoys. Based on Percolator-derived q-values, allowed peptide FDR was set to 1% or less. Grouping of peptides into proteins was performed with strict parsimony by the Protein Grouping node. Protein-level FDR was calculated by the Protein FDR Validator Node, where an FDR of 1% or less was considered High Confidence.

TMTpro reporter signals were integrated with a tolerance of 20 ppm using the “Most Confident Centroid” method in the Reporter Ions Quantifier node. Only MS3 spectra with an average signal-to-noise ratio (S/N) of 10 or more were considered for the peptide-to-protein quantification rollup. S/N values were used as the effective quantification values in each channel. Additionally, a co-isolation threshold of max. 70% and a cutoff for minimum required SPS-masses matched to the identified peptide of 65% were applied.

Normalization and Scaling were performed based on five pre-selected carboxylases (P11498, Q13085, Q96RQ3, P05165, O00763). First, S/N values from all PSMs of a given peptide are summed. To obtain protein level abundances, abundance values from all respective peptides are summed for each channel, resulting in 16 summed abundance values for each protein accession. Abundances belonging to reference accessions (carboxylases) are summed again channel-wise and the resulting 16 values divided by their largest member, resulting in 16 correction factors. For each of the 16 channels, every protein-level abundance value is divided by its respective correction factor, resulting in normalized protein abundances. Values from the previous step are normalized protein-wise, starting with calculating the mean abundance across all channels for a given protein. Each row-mean is then divided by 100 to obtain a scaling factor, and each value in the respective row is divided by that row’s scaling factor. Of note, this scaling method standardizes comparisons between different channels of the same protein but prohibits comparisons across different proteins within the same channel. Lastly, the mean of all channels belonging to the same sample group is obtained, allowing for the calculation of grouped abundance ratios between treatment groups for a given protein, as well as p-values via two-sample ANOVA test. Multiple-testing adjusted p-values (Benjamini Hochberg FDR method) are reported as well, the usage of raw or adjusted p-values is indicated throughout the study.[11]

2.13 Cellular thermal shift assay (CETSA)

The TRPV2 protein stability was measured using the CETSA assay by treating cell lysate with compound at desired concentrations. Briefly, MDA-MB-231 cells were plated in T75 flasks at 60% confluency the day before collection. The cells were then washed with PBS and harvested in 1 mL of a lysis solution (50 mM Tris-HCl, pH 7.5, 5 % glycerol, 100 mM NaCl, 2.5 mM $MgCl_2$, 0.2 % NP40, and protease inhibitor (Roche)). After incubation on ice for 30 min, the lysate was centrifuged at 14,000 g for 20 min at 4 °C, supernatants were transferred to new tubes, incubated with the different drugs including HKC22, HKC54 or Vehicle at 10 µM for 30 min on ice. Then the treated lysate was aliquoted into PCR tubes (80 µL per tube) and heated individually on a thermal cycler at different temperatures from 45 - 80 °C for 6 min and then cooled at room temperature for 3 min. Following centrifugation at 14,000 g for 40 min at 4 °C, supernatants were transferred to new tubes and stored at - 80 °C until immunoblotting was performed.

CETSA samples were separated by sodium dodecyl sulfate-polyacrylamide gel electrophoresis, and immunoblotting was performed using a polyclonal anti-TRPV2 antibody (Abcam, ab272862, 1: 500 dilution), GAPDH (ABclonal, AC036, 1:1000 dilution), beta Actin (Santa Cruz, sc-47778, 1:1000 dilution), anti-rabbit IgG, HRP-linked antibody (Abcam, ab6721, 1:5000 dilution), anti-mouse IgG, HRP-linked antibody (Abcam, ab205719, 1:5000 dilution). The intensities of the bands were quantified using ImageJ.

2.14 Wound healing assay in PANC-1 cells

A single scratch was made with a p200 tip through each well of a confluent 24 well plate of PANC-1 cells in DMEM 2.5% FBS. Wells were treated with 5 or 10 µM PL, HKC22 or DMSO vehicle and the scratch area was measured after 8 h. Brightfield images were acquired using an EVOS M5000 microscope. Images were analysed using ImageJ and a plugin developed specifically for analysing wound healing assay scratch areas.[12]

2.15 Wound healing assay in U-251 and U-251 TRPV2 KD cells

2 x 10^5 cells/well of U251 WT or U251 TRPV2-KD cells were seeded into 24-well plate in DMEM with 10 % FBS, and incubated for 24h. A single scratch was then made with a p200 tip through each well, and wells were treated with 5 or 10 µM PL, HKC22, HKC54 or DMSO vehicle in DMEM with 2.5% FBS and the scratch area was measured after 20, 40 or 60 h. Brightfield images were acquired using ZEISS Celldiscoverer 7 with LSM 900 microscope. Images were analysed using Image/Fiji with the scratch assay plugin.

2.16 Computational methods

2.16.1 Protein visualisation and figure creation software

Protein structures from the PDB[13] were visualised using PyMOL or BioRender. Figures were created using either PyMOL or BioRender.

2.16.2 Molecular docking of piperlongumine and derivatives

Molecular docking of HKC22 and HKC54 was performed using Flare™ by Cresset by docking the compounds into the PL-bound TRPV2 cryo-EM structure (PDB: 6WKN) using a box size of 6 Å. The lowest energy binding poses are presented.

2.16.3 Molecular dynamics simulations of HKC22 bound to TRPV2

The calculations were carried out with AMBER 22 package[14] implemented with ff14SB,[15] GAFF[16] and Lipid14[17] force fields. Bilayers of 1,2-dioleoyl-sn-glycero-3-phosphocholine lipid, together with the cryo-EM structure of the complex TRPV2/PL (PDB entry: 6WKN), TIP3P water molecules and KCl to neutralize the system, were constructed using CHARMM Membrane Builder GUI[18] and converted to Lipid14 PDB format with the charmmlipid2amber.x script included in AMBER. Parameters for ligands were generated with the antechamber module of AMBER and GAFF force field. Partial charges of the ligand were calculated using AM1-BCC[19] method as implemented in antechamber.

The full system was minimized for 10000 steps, of which the first 5000 steps used the steepest descent method and the remaining steps used the conjugate gradient method.[20] The system was then heated from 0 K to 100 K using Langevin dynamics[21] for 5 ps at constant volume, with weak restraints on the lipid (force constant 10 kcal·mol^{-1}·Å^{-2}). Following this, the volume was allowed to change freely, and the temperature increased to 300 K with a Langevin collision frequency of γ=1.0 ps^{-1}, and anisotropic Berendsen regulatione[22] (1 atm) with a time constant of 2 ps for 100 ps. The same weak restraint of 10 kcal·mol^{-1}·Å^{-2} was maintained on the lipid molecules. Constant pressure (1 atm) and constant temperature (300K) (NPT) runs (500 ps $\times$ 10) were then performed. Bonds involving hydrogen were constrained using the SHAKE algorithm, allowing a 2-fs time step. Structural data was recorded every 10 ps. PME was used to treat all electrostatic interactions with a real space cutoff of 10 Å. A long-range analytical dispersion correction was applied to the energy and pressure. Temperature (300 K) was controlled by the Langevin thermostat, with a collision frequency of γ=1.0 ps^{-1}. Pressure was regulated by the anisotropic Berendsen method (1 atm) with a pressure relaxation time of 1.0 ps. Production trajectories were then run for 300 ns.

2.17 Maximum tolerated dose (MTD) test

MTD study was performed at the Instituto de Medicina Molecular João Lobo Antunes (IMM, Lisbon) with strict adherence to the Portuguese Law (Portaria 1005/92) and the European Guideline 86/609/EEC. The Federation of European Laboratory Animal Science Associations guidelines and recommendations concerning laboratory animal welfare were followed. Animal experiments were approved by the Portuguese official veterinary department for welfare licensing – Direção Geral de Alimentação e Veterinária (DGAV) and the IMM Animal Ethics Committee (authorization AWB_2021_03_GB_TargCancerDrugs). 8-week-old female BALB/c mice (purchased from Charles River) were intraperitoneal (IP) injected with PL, HKC22, HKC54 at 1, 10 or 25 mg/kg or Vehicle control every other day, in a total of five doses. Body weight was monitored until Day 14 (five days after last treatment).

2.18 In vivo metastasis model

The metastasis study was conducted in the In-vivo pharmacology facility at Syngene International Ltd., with the compliance as per the regulations of the Committee for Control and Supervision of Experiments on Animals (CCSEA), Government of India and Association for Assessment and Accreditation of Laboratory Animal Care (AAALAC) guidelines. The 'Form B' for carrying out animal experimentation was reviewed and approved by the Institutional Animal Ethics Committee.

Preparation of tumor cells. All procedures were performed in laminar flow hood following sterile techniques. RPMI-1640 media along with 10% FBS, 1% Antibiotic-Antimycotic and Blasticidin 8 μg/mL were used for growing the cells. Cancer cells with viability of >90 % were chosen for the study. 2.5 X 10^5 cells were re-suspended in 200 μL of serum free media and placed in ice.

Tumor cell implantation. Female Balb/c mice (7-9 weeks old) were used for the present investigation. On day -1, the cells were injected into the animals by intravenous tail vein route (200μL/animal).

Randomization. On day 0, animals were randomized based on the body weight and dosing initiated with test compounds. Immediately after randomization, the animals were assigned a permanent number. Cages were identified by cage cards indicating the study number, group number, sex, dose, cage number, number of animals.

Formulation preparation, dose volume of test compounds. For all test items, formulation: Compound was weighed and taken into mortar and pestle. First compound was triturated with tween 80 (10% of total volume). Then PBS (90% of total volume) was added slowly with

continuous trituration. Then the formulation was ultra-sonicated for 3 cycles (15 seconds each). Dose volume was maintained at 10mL/kg for dosing.

Termination and sample collection. At the termination, lungs samples were collected and weighed. The lung samples were then placed in Bouin's solution and then the manual counting was performed for nodule counts using Stereo microscope.

REFERENCES

1. Pangborn, A.B., Giardello, M.A., Grubbs, R.H., Rosen, R.K., and Timmers, F.J. (1996). Safe and Convenient Procedure for Solvent Purification. Organometallics *15*, 1518–1520. https://doi.org/10.1021/om9503712.

2. Meegan, M.J., Nathwani, S., Twamley, B., Zisterer, D.M., and O'Boyle, N.M. (2017). Piperlongumine (piplartine) and analogues: Antiproliferative microtubule-destabilising agents. European Journal of Medicinal Chemistry *125*, 453–463. https://doi.org/10.1016/J.EJMECH.2016.09.048.

3. Adams, D.J., Dai, M., Pellegrino, G., Wagner, B.K., Stern, A.M., Shamji, A.F., and Schreiber, S.L. (2012). Synthesis, cellular evaluation, and mechanism of action of piperlongumine analogs. Proceedings of the National Academy of Sciences of the United States of America *109*, 15115–15120. https://doi.org/10.1073/pnas.1212802109.

4. Zazeri, G., Povinelli, A.P.R., Le Duff, C.S., Tang, B., Cornelio, M.L., and Jones, A.M. (2020). Synthesis and Spectroscopic Analysis of Piperine- and Piperlongumine-Inspired Natural Product Scaffolds and Their Molecular Docking with IL-1β and NF-κB Proteins. Molecules *25*, 2841. https://doi.org/10.3390/molecules25122841.

5. Toyota, Y., Yoshioka, H., Sagimori, I., Hashimoto, Y., and Ohgane, K. (2020). Bisphosphonate esters interact with HMG-CoA reductase membrane domain to induce its degradation. Bioorganic and Medicinal Chemistry *28*, 115576. https://doi.org/10.1016/j.bmc.2020.115576.

6. Dubin, A.E., Murthy, S., Lewis, A.H., Brosse, L., Cahalan, S.M., Grandl, J., Coste, B., and Patapoutian, A. (2017). Endogenous Piezo1 can confound mechanically-activated channel identification and characterization. Neuron *94*, 266-270.e3. https://doi.org/10.1016/j.neuron.2017.03.039.

7. Rodrigues, T., Sieglitz, F., Somovilla, V.J., Cal, P.M.S.D., Galione, A., Corzana, F., and Bernardes, G.J.L. (2016). Unveiling (−)-Englerin A as a Modulator of L-Type Calcium Channels. Angewandte Chemie International Edition *55*, 11077–11081. https://doi.org/10.1002/anie.201604336.

8. Mathivanan, S., Devesa, I., Changeux, J.-P., and Ferrer-Montiel, A. (2016). Bradykinin Induces TRPV1 Exocytotic Recruitment in Peptidergic Nociceptors. Frontiers in Pharmacology *7*.

9. Devesa, I., Ferrándiz-Huertas, C., Mathivanan, S., Wolf, C., Luján, R., Changeux, J.-P., and Ferrer-Montiel, A. (2014). αCGRP is essential for algesic exocytotic mobilization of TRPV1 channels in peptidergic nociceptors. Proceedings of the National Academy of Sciences *111*, 18345–18350. https://doi.org/10.1073/pnas.1420252111.

10. Theodoropoulos, P.C., Wang, W., Budhipramono, A., Thompson, B.M., Madhusudhan, N., Mitsche, M.A., McDonald, J.G., De Brabander, J.K., and Nijhawan, D. (2020). A Medicinal Chemistry-Driven Approach Identified the Sterol Isomerase EBP as the Molecular Target of TASIN Colorectal Cancer Toxins. J Am Chem Soc *142*, 6128–6138. https://doi.org/10.1021/jacs.9b13407.

11. Offensperger, F., Tin, G., Duran-Frigola, M., Hahn, E., Dobner, S., Ende, C.W.A., Strohbach, J.W., Rukavina, A., Brennsteiner, V., Ogilvie, K., et al. (2024). Large-scale chemoproteomics expedites ligand discovery and predicts ligand behavior in cells. Science *384*, eadk5864. https://doi.org/10.1126/science.adk5864.

12. Suarez-Arnedo, A., Torres Figueroa, F., Clavijo, C., Arbeláez, P., Cruz, J.C., and Muñoz-Camargo, C. (2020). An image J plugin for the high throughput image analysis of in vitro scratch wound healing assays. PLoS One *15*, e0232565. https://doi.org/10.1371/journal.pone.0232565.

13. Berman, H.M., Westbrook, J., Feng, Z., Gilliland, G., Bhat, T.N., Weissig, H., Shindyalov, I.N., and Bourne, P.E. (2000). The Protein Data Bank. Nucleic Acids Research *28*, 235–242. https://doi.org/10.1093/nar/28.1.235.

14. Case, D.A., Aktulga, H.M., Belfon, K., Cerutti, D.S., Cisneros, G.A., Cruzeiro, V.W.D., Forouzesh, N., Giese, T.J., Götz, A.W., Gohlke, H., et al. (2023). AmberTools. J. Chem. Inf. Model. *63*, 6183–6191. https://doi.org/10.1021/acs.jcim.3c01153.

15. Maier, J.A., Martinez, C., Kasavajhala, K., Wickstrom, L., Hauser, K.E., and Simmerling, C. (2015). ff14SB: Improving the Accuracy of Protein Side Chain and Backbone Parameters from ff99SB. J. Chem. Theory Comput. *11*, 3696–3713. https://doi.org/10.1021/acs.jctc.5b00255.

16. Wang, J., Wolf, R.M., Caldwell, J.W., Kollman, P.A., and Case, D.A. (2004). Development and testing of a general amber force field. Journal of Computational Chemistry *25*, 1157–1174. https://doi.org/10.1002/jcc.20035.

17. Dickson, C.J., Madej, B.D., Skjevik, Å.A., Betz, R.M., Teigen, K., Gould, I.R., and Walker, R.C. (2014). Lipid14: The Amber Lipid Force Field. J. Chem. Theory Comput. *10*, 865–879. https://doi.org/10.1021/ct4010307.

18. Jo, S., Lim, J.B., Klauda, J.B., and Im, W. (2009). CHARMM-GUI Membrane Builder for Mixed Bilayers and Its Application to Yeast Membranes. Biophys J *97*, 50–58. https://doi.org/10.1016/j.bpj.2009.04.013.

19. Jakalian, A., Jack, D.B., and Bayly, C.I. (2002). Fast, efficient generation of high-quality atomic charges. AM1-BCC model: II. Parameterization and validation. J Comput Chem *23*, 1623–1641. https://doi.org/10.1002/jcc.10128.

20. Numerical recipes art scientific computing 3rd edition | Numerical recipes | Cambridge University Press https://www.cambridge.org/gb/universitypress/subjects/mathematics/numerical-recipes/numerical-recipes-art-scientific-computing-3rd-edition?format=HB&utm_source=shortlink&utm_medium=shortlink&utm_campaign=numericalrecipes.

21. Pastor, R.W., Brooks, B.R., and Szabo, A. (1988). An analysis of the accuracy of Langevin and molecular dynamics algorithms. Molecular Physics *65*, 1409–1419. https://doi.org/10.1080/00268978800101881.

22. Berendsen, H.J.C., Postma, J.P.M., van Gunsteren, W.F., DiNola, A., and Haak, J.R. (1984). Molecular dynamics with coupling to an external bath. The Journal of Chemical Physics *81*, 3684–3690. https://doi.org/10.1063/1.448118.
